# Narcolepsy as an immune-associated hypothalamic encephalopathy: orexin dysfunction and implications for precision sleep medicine

**DOI:** 10.3389/fpsyt.2026.1799520

**Published:** 2026-04-02

**Authors:** Oscar Arias-Carrión, Emmanuel Ortega-Robles, Patricia Romano, Carlos Pineda

**Affiliations:** 1 División de Neurociencias Clínica, Instituto Nacional de Rehabilitación Luis Guillermo Ibarra Ibarra, Mexico City, Mexico; 2 Tecnologico de Monterrey, Escuela de Medicina y Ciencias de la Salud, Mexico City, Mexico; 3 División de Investigación en Ingeniería Médica, Instituto Nacional de Rehabilitación Luis Guillermo Ibarra Ibarra, Mexico City, Mexico; 4 División de Reumatología, Instituto Nacional de Rehabilitación Luis Guillermo Ibarra Ibarra, Mexico City, Mexico

**Keywords:** autoimmunity, hypothalamic circuits, narcolepsy, orexin/hypocretin, precision sleep medicine, sleep-wake regulation

## Abstract

Narcolepsy can no longer be adequately conceptualized by excessive sleepiness and cataplexy. It is increasingly recognized as a multisystem hypothalamic encephalopathy, rooted in the selective loss or dysfunction of orexin neurons, yet extending across motor, psychiatric, metabolic, and autonomic domains. Over the past two decades, convergent genetic, neuropathological, and immunological evidence has positioned narcolepsy type 1 as increasingly consistent with the spectrum of immune-mediated neurological diseases while challenging the validity of current classifications that hinge on cataplexy or multiple sleep latency testing. Borderland phenotypes, variable orexin biology, and post-infectious or secondary forms underscore the limitations of rigid categorical nosologies and support a spectrum-based framework. Advances in immunology, imaging, and systems biology highlight the limitations of purely symptomatic treatment and support the exploration of mechanism-based interventions, including orexin receptor agonism, immune-targeted strategies in early disease, and regenerative or circuit-repair approaches. In this narrative review, based on literature identified through searches of PubMed, Web of Science, and Scopus through December 2025, we synthesize evidence across epidemiology, pathophysiology, diagnosis, and therapy, and propose an integrative clinical algorithm that moves beyond categorical diagnoses toward a phenotype–biomarker–mechanism stratification model. We suggest that narcolepsy should no longer be considered a rare curiosity of sleep medicine but rather a model disorder illuminating the vulnerability of hypothalamic circuits and the complex interplay between sleep, emotion and immunity.

## 1 Introduction

Narcolepsy should no longer be described as a disorder of excessive sleepiness punctuated by cataplexy. It is emerging as a model disease at the intersection of neuroimmunology, hypothalamic biology, and systems neuroscience (1). Its study has forced a shift away from reductionist views of sleep disorders toward broader questions: How do discrete hypothalamic circuits sustain wakefulness? How do immune processes selectively target a population of ~70,000 neurons while sparing neighboring cell groups?, and how does destabilization of REM sleep control reshape emotion, cognition, metabolism, and autonomic physiology?

Historically dismissed as a curiosity of sleep medicine, narcolepsy is now recognized as a brain disorder whose roots extend from genetics and immune regulation to epigenetic and environmental factors (1, 2). The selective loss—or functional silencing—of orexin-producing neurons represents a well-recognized example of circuit-specific vulnerability in the human brain (1). Although anatomically circumscribed, this lesion produces a multisystem syndrome that encompasses motor collapse, hallucinations, disrupted vigilance, psychiatric comorbidities, metabolic dysregulation, and autonomic dysfunction, reflecting both the broad projections of orexin neurons and the fragility of interconnected hypothalamic–limbic–brainstem networks (2).

Convergent immunogenetic and mechanistic evidence support an immune-mediated pathophysiology. Autoreactive CD4^+^ and CD8^+^ T cells recognize antigens from orexin neurons (3, 4), and molecular mimicry between influenza antigens and orexin peptides provides a plausible trigger for immune tolerance breakdown (5). Epidemiological observations further underscore this link: incidence varies across populations and has shown abrupt, geographically specific surges associated with H1N1 infection and vaccination campaigns (6).

At the same time, narcolepsy unsettles classical diagnostic categories. Cataplexy, long considered its defining hallmark, is not always present. Cerebrospinal fluid orexin deficiency—once thought pathognomonic—may be absent in borderline or secondary phenotypes, in part because conventional immunoassays may incompletely capture orexin-related fragments rather than intact peptide (7). Contemporary nosology reflects this heterogeneity: the International Classification of Sleep Disorders, Third Edition, Text Revision (ICSD-3-TR, 2023) refines and clarifies the 2014 ICSD-3 criteria by updating diagnostic terminology, consolidating guidance on biomarker use (including cerebrospinal fluid orexin thresholds), and reinforcing distinctions among central disorders of hypersomnolence. It further emphasizes phenotype- and biomarker-informed diagnosis, highlighting the limitations of rigid categorical frameworks (**Table 1**) (8, 9).

**Table 1:** Clinical phenotypes of narcolepsy.

Clinical phenotype	Defining features	Proportion of cases (%)	Notes/associations
Narcolepsy with typical cataplexy and all biological markers	Classic phenotype with unequivocal cataplexy; presence of HLA-DQB1*06:02, ≥2 SOREMPs, and low or undetectable CSF orexin-A	50–80	Represents the best-defined entity (NT1); usually sporadic; often associated with immune-mediated orexin loss
Narcolepsy with typical cataplexy but without biological markers	Cataplexy present, but absence of HLA-DQB1*06:02 and/or normal CSF orexin-A levels; SOREMPs variable	5–10	Often familial or secondary (for example, structural hypothalamic lesions); rare, sometimes stable over time
Narcolepsy without cataplexy but with some biological markers	No cataplexy; at least one marker present (HLA-DQB1*06:02 positivity, SOREMPs, or borderline orexin deficiency)	18–34	Includes NT2 and “borderland” phenotypes; some patients progress to NT1 over months to decades
Narcolepsy without cataplexy and without biological markers	EDS and fragmented sleep; absence of cataplexy, SOREMPs, orexin deficiency, or HLA association	Unknown (<5)?	Controversial; often overlaps with idiopathic hypersomnia; might represent non-REM narcolepsy
Secondary narcolepsy	Narcolepsy-like syndrome following hypothalamic or brainstem damage (for example, neurosarcoidosis, neuromyelitis optica, multiple sclerosis, traumatic brain injury)	Rare (<1)	Clinical profile often atypical; orexin deficiency may or may not be present
Hereditary narcolepsy (“narcolepsy-plus” syndromes)	Narcolepsy accompanied by additional neurological features (for example, deafness, cerebellar ataxia, polyneuropathy, metabolic syndromes)	Very rare	Usually familial; mutations in immune or orexin-related pathways are sometimes reported
Pediatric narcolepsy phenotype	Onset in childhood; often characterized by rapid weight gain, facies cataplectica, behavioral disturbances (hyperactivity, aggression, depression), and early puberty	10–15% of all cases of narcolepsy	Clinical manifestations evolve with age; biomarkers may be inconsistent in early years
Narcolepsy with atypical or psychogenic cataplexy (“pseudocataplexy”)	Episodes resembling cataplexy but triggered by atypical emotions, prolonged duration, and preserved reflexes often coexist with psychiatric comorbidity	Unclear, probably under-recognized	Represents a diagnostic challenge; it requires videography and neurophysiological testing

NT1, narcolepsy type 1; NT2, narcolepsy type 2; CSF, cerebrospinal fluid; HLA, human leukocyte antigen; EDS, excessive daytime sleepiness; REM, rapid eye movement; SOREMPs, sleep-onset REM periods.

This reconceptualization carries therapeutic implications. If narcolepsy is an immune-mediated hypothalamic encephalopathy, then purely symptomatic approaches are insufficient. Mechanism-directed strategies now include orexin-2 receptor agonists that directly address the neurotransmitter deficit—first demonstrated with TAK-994 (efficacious but halted due to hepatotoxicity) and then with oveporexton (TAK-861), which improved wakefulness and cataplexy without liver toxicity in phase 2 trials (10, 11). Parallel experimental work suggests the feasibility of circuit repair via orexin-cell transplantation to restore motor–arousal coupling and reduce cataplexy in animal models (12, 13).

In this narrative review, we synthesize evidence on epidemiology, clinical spectrum, pathological and genetic underpinnings, and emerging immunological mechanisms. We discuss advances in diagnostic frameworks and therapeutics and propose a revised clinical framework that moves beyond categorical diagnoses toward precision sleep medicine based on phenotype–biomarker–mechanism integration. We argue that narcolepsy should be viewed not merely as a rare sleep disorder, but as a model disease illuminating fundamental principles of hypothalamic circuit fragility and neuroimmune interaction.

## 2 Methodology

This article was conceived as a narrative review, given the breadth of the topic, the large and rapidly evolving body of literature, and the aim of providing an integrative conceptual framework rather than systematically aggregating quantitative outcomes. The primary objective was to synthesize current knowledge across immunology, genetics, pathology, systems neuroscience, diagnosis, and therapeutics, and to outline future translational directions.

For foundational and conceptual sections, literature was selected based on relevance, impact, recency, and methodological robustness, with emphasis on high-quality reviews, consensus statements, large cohort studies, and pivotal mechanistic investigations.

For the therapeutic section and the evidence summary, a structured literature search was conducted in PubMed/MEDLINE, Web of Science, and Scopus for preclinical and clinical studies published in English up to December 2025. The population of interest included pediatric and adult individuals diagnosed with narcolepsy type 1 or type 2, as well as studies referring to cataplexy, excessive daytime sleepiness, or orexin deficiency. The intervention component focused on pharmacological and mechanism-targeted therapies, including sodium oxybate, low-sodium oxybate, once-nightly sodium oxybate, pitolisant, solriamfetol, modafinil, armodafinil, methylphenidate, amphetamines, and selective orexin receptor agonists such as TAK-861 (oveporexton), TAK-925 (danavorexton), and TAK-994. The comparison framework included placebo-controlled designs, active comparators, standard-of-care treatments, and baseline pretreatment conditions, with emphasis on randomized controlled trials and phase 2 or phase 3 clinical studies. Outcomes of interest comprised validated efficacy and safety endpoints, including Epworth Sleepiness Scale scores, Maintenance of Wakefulness Test latency, weekly cataplexy frequency, sleep-onset REM periods, quality-of-life measures, and adverse event profiles.

Eligible studies included randomized controlled trials, prospective clinical trials, major observational studies, and high-quality systematic reviews relevant to therapeutic efficacy and safety. Preclinical studies were included when they directly informed mechanism-based treatment strategies. Case reports, studies with insufficient methodological detail, duplicate reports, or publications lacking primary outcome data were excluded at the authors’ discretion.

As this was not designed as a systematic review, formal risk-of-bias assessment tools were not applied. Certainty assessments were derived from expert consensus using a GRADE-informed conceptual approach. Given the breadth and heterogeneity of the available literature, judgments were based on study design hierarchy, consistency and reproducibility of findings across trials, magnitude and clinical relevance of effects, and the presence of regulatory-level evidence (e.g., pivotal phase 3 trials and approvals), rather than on formal upgrading or downgrading algorithms.

## 3 Epidemiology

The prevalence of narcolepsy varies considerably across populations. In Europe and North America, estimates range from 200 to 500 cases per million individuals (14, 15). More recent claims- and survey-based studies suggest that diagnosed prevalence in high-income countries most commonly clusters between 30 and 50 per 100,000 individuals (300–500 per million), although substantial methodological heterogeneity persists (16–18). In a recent U.S. general population study using structured interviews, the estimated prevalence of narcolepsy was 37.7 per 100,000, with an incidence of 2.6 per 100,000 person-years (17). Similarly, a large Japanese claims-based study (JMDC database; >6 million individuals) reported an age–sex standardized prevalence of 37.5 per 100,000 and an incidence of 5.1 per 100,000 person-years (18). A recent global meta-analysis confirmed marked between-study heterogeneity, largely driven by differences in case definitions (ICD vs ICSD), ascertainment strategies (claims vs community surveys), age structure, and post-2009 pandemic effects (16, 19). The epidemiology remains imprecise, in part because of diagnostic uncertainty, especially within the so-called narcoleptic borderland, most notably narcolepsy type 2 (NT2), and because administrative databases rarely distinguish NT1 from NT2 or capture undiagnosed cases (16, 18).

The prevalence is reported to be highest in Japan, reaching 1,600 per million, although methodological limitations challenge the reliability of this estimate. These very high figures derive primarily from older questionnaire- and interview-based surveys that rely on self-reported symptoms and limited diagnostic verification and are likely to reflect overestimation (18, 20). In contrast, contemporary Japanese claims-based analyses report substantially lower, internationally comparable estimates (approximately 35–45 per 100,000), depending on the case definition (18). In contrast, Jewish and Arabic populations show the lowest prevalence (2–40 per million) (21). Marked geographic variation has also been described within Asia and Europe, partly paralleling the distribution of HLA-DQB1*06:02 and possibly reflecting gene–environment interactions (16, 20). Some series suggest a modest male predominance (21). Recent large administrative datasets also report a slight male predominance in both prevalence and incidence, although this may be influenced by database composition and “healthy worker” effects (18). Such heterogeneity underscores the likely interplay of genetic and environmental influences. Reports of increased mortality remain inconclusive (22, 23).

Narcolepsy typically presents during adolescence, with a secondary peak of onset in the third decade of life (24). Contemporary incidence data confirm that rates are highest in the 10–19 and 20–29 year age groups, with a subsequent decline in later adulthood (16, 18). In 10–15% of cases, onset occurs before the age of 10 (25). Until recently, pediatric narcolepsy has been under-recognized and under-studied (26, 27). Population-based pediatric studies report pre-2009 incidence rates generally below 1 per 100,000 person-years in Europe and North America, but marked transient increases were observed after the 2009 H1N1 pandemic in several countries, particularly in vaccinated children in Northern Europe (16, 20). In Finland and Sweden, pediatric incidence increased more than tenfold in the immediate post-pandemic period, highlighting the impact of environmental triggers on genetically susceptible individuals (20). Clinical trajectories are diverse: symptoms may appear abruptly following a trigger such as infection, vaccination, stress, or head trauma; evolve insidiously with an indistinct onset; or progress stepwise, with years separating the emergence of individual features (28). Such variability suggests distinct pathophysiological mechanisms (28).

Cataplexy most often coincides with the onset of excessive daytime sleepiness (EDS). In a cohort of 1,099 patients, cataplexy developed simultaneously with EDS in 49%, followed EDS in 43%, and preceded it in only 8% (29). The typical interval between EDS and cataplexy is fewer than three years, but in extreme cases, it spans several decades (29, 30).

Narcolepsy without cataplexy may remit spontaneously (31, 32), whereas narcolepsy with cataplexy rarely does; to date, remission has been reported only once, following immunotherapy initiated soon after onset (33). Over time, symptoms such as EDS and cataplexy often become less disabling, possibly owing to compensatory coping strategies and pharmacological treatment. Nevertheless, substantial diagnostic delay—often approaching one to two decades in some series—continues to distort epidemiologic estimates and likely contributes to under-recognition in older adults and minority populations (19).

## 4 Etiology

Early case reports from the beginning of the 20th century noted that narcolepsy sometimes followed infectious or traumatic events, suggesting perturbations of immune or neural stability (34). By the 1980s, the strong and reproducible association with HLA alleles, particularly DQB1*06:02, had firmly established an immune-mediated contribution to disease susceptibility (35), a concept reinforced by more recent genome-wide and immunogenetic studies (1, 36).

The subsequent demonstration of reduced cerebrospinal fluid (CSF) orexin-A levels in many patients, coupled with findings of selective loss of orexin-producing neurons in the lateral hypothalamus, redirected etiological models toward targeted immune-mediated disruption of these neurons (3, 4). Orexin-A and B, acting via OX_1_R and OX_2_R, sustain excitatory drive across cortical, limbic, diencephalic and brainstem circuits (37, 38). Their deficiency destabilizes sleep-wake boundaries, producing prominent REM intrusion into wakefulness, cataplexy and vivid dream phenomena at transitional states (39).

Nevertheless, several discrepancies challenge a unidimensional model. Patients without cataplexy or at early disease stages may retain normal CSF orexin (hypocretin-1) levels, while individuals with structural hypothalamic lesions and reduced orexin may not develop narcoleptic symptoms (40). Intermediate CSF orexin levels further complicate interpretation (41, 42). These observations demonstrate that orexin deficiency, although central, is neither universally necessary nor sufficient to explain the full clinical spectrum, supporting the existence of multiple etiological forms of narcolepsy (**Table 2**).

**Table 2:** Etiological forms of narcolepsy.

Etiological form	Defining features	Proportion of cases* (%)	Notes/associations
Sporadic	Most frequent form; no family history; usually associated with HLA-DQB1*06:02; often preceded by environmental triggers (e.g., infection, vaccination, head trauma); strong evidence for autoimmune-mediated loss of orexin neurons	>90	Typically NT1 with cataplexy; orexin deficiency is present in most cases; it represents the prototypical form studied in large cohorts
Familial	Narcolepsy affecting ≥2 family members; often variable penetrance; may present with or without cataplexy; orexin levels and HLA associations inconsistent	<5	Suggests contribution of rare heritable risk variants; usually autosomal but not Mendelian inheritance; may overlap with borderline phenotypes
Secondary (symptomatic)	Occurs after hypothalamic or brainstem injury, inflammatory or demyelinating disease (e.g., multiple sclerosis, neurosarcoidosis, neuromyelitis optica), tumors, vascular lesions, or traumatic brain injury	<5	Clinical course often atypical; may coexist with other neurological deficits; orexin deficiency variable depending on site and severity of injury
Hereditary “narcolepsy-plus” syndromes	Narcolepsy occurring as part of multisystem or syndromic disorders, often with additional neurological features (e.g., deafness, cerebellar ataxia, neuropathy, metabolic or genetic syndromes)	<1	Very rare; linked to mutations in orexin, immune, or metabolic pathways; provides insight into developmental and systemic contributions to narcolepsy
Post-infectious or post-vaccination (emerging category)	Narcolepsy temporally associated with infections (influenza A/H1N1, streptococcal infection, encephalitis) or, rarely, vaccinations (notably Pandemrix^®^ H1N1)	Unknown (geographically variable; peaks reported after 2009–2010 pandemic)	Evidence supports molecular mimicry and immune cross-reactivity as plausible mechanisms, providing a useful model for understanding how environmental triggers may interact with genetic susceptibility.
Epigenetic and environmental forms (borderland concept)	Cases in which early-life exposures (season of birth, toxins, perinatal insults) or persistent epigenetic modifications influence narcolepsy risk, even in the absence of family history	Not precisely quantified	Emerging area of research; likely interacts with sporadic and familial forms; helps explain incomplete penetrance of genetic susceptibility

*Proportions are approximate and may vary by cohort, geography, and ascertainment method; categories are not mutually exclusive and may overlap. NT1, narcolepsy type 1; HLA, human leukocyte antigen.

An increasingly persuasive multi-hit model proposes that genetic predisposition, environmental exposures and immune activation converge to disrupt orexin neurons (36, 43). HLA alleles remain the strongest risk markers, with DQB1*06:02 present in the overwhelming majority of individuals with narcolepsy type 1 (NT1), but this background alone rarely produces disease. Additional loci—including *TCRα*, *P2RY11*, and *CTSC*—and emerging epigenetic modifiers likely influence immune tolerance and neuronal vulnerability (44). Residual orexin neurons may persist in some patients but remain functionally silent through epigenetic repression, although robust validation in human tissue is still pending.

Evidence accumulated during the past five years sharply intensifies the immune narrative. Autoreactive CD4^+^ and CD8^+^ T cells that recognize orexin peptides have been identified in both NT1 and NT2 types of narcolepsy (3–5). Recent postmortem studies reveal a striking accumulation of CD4^+^ T cells within the orexin neuronal field compared with neighboring hypothalamic regions or control brains, demonstrating selective immune infiltration (45, 46). In parallel, experimental models show that loss of orexin function enhances microglial activation and inflammatory signaling, suggesting that orexin neurons play an active role in neuroimmune homeostasis and become selectively vulnerable when tolerance fails (46).

Explaining clinical heterogeneity requires integrating neuronal and network dimensions (47). In patients with preserved orexin concentrations, functional suppression—mediated by receptor downregulation, synaptic dysfunction or cytokine-induced inhibition—may precede frank cell loss. Conversely, rare cases of orexin depletion without narcoleptic manifestations imply that intact downstream circuits or compensatory plasticity can buffer partial hypothalamic dysfunction (48). Symptom emergence, therefore, appears to reflect both orexin cell survival and the resilience of distributed arousal networks that maintain stability under stress.

**Figure 1:**
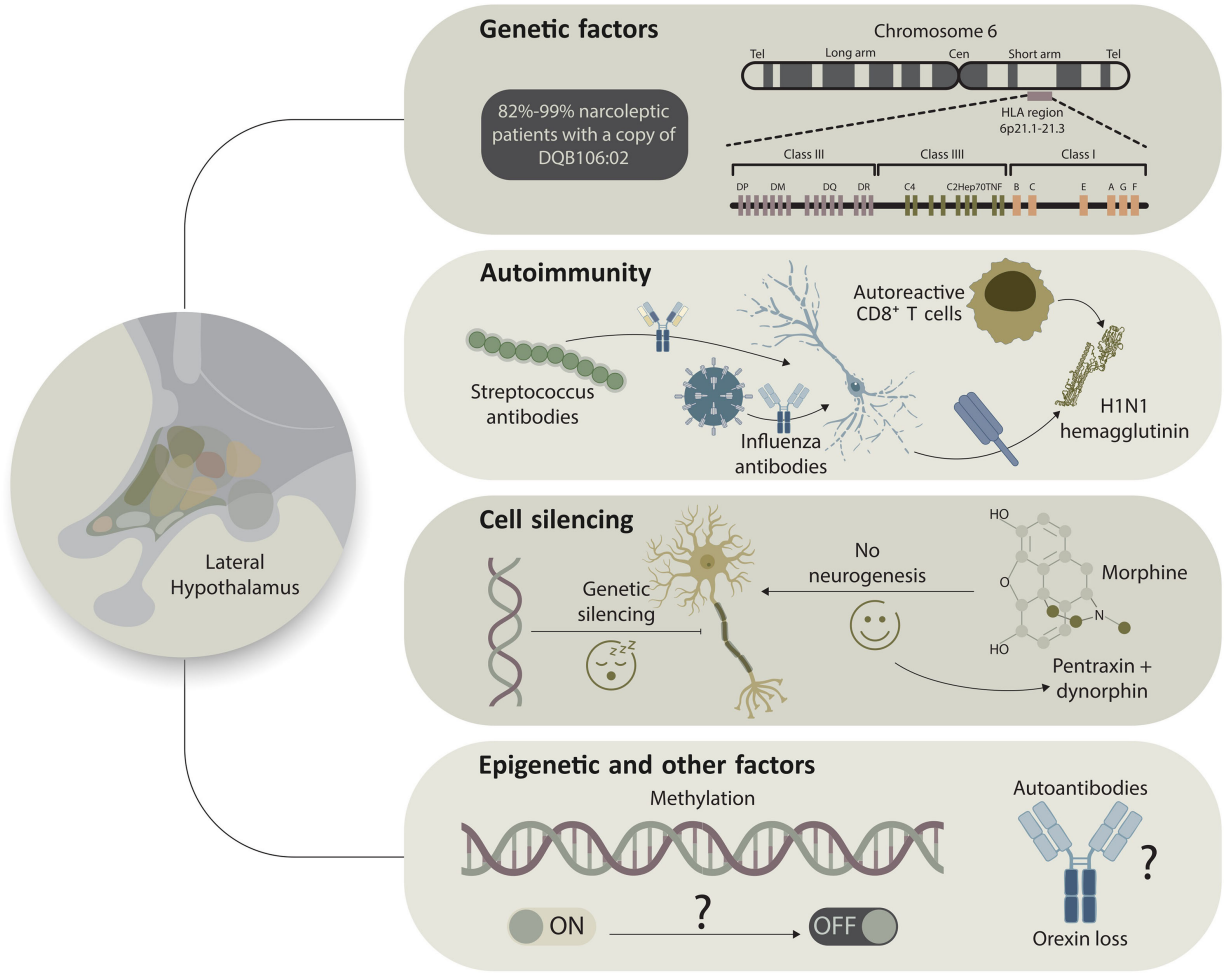
Converging genetic, immune, epigenetic, and cellular mechanisms in the pathogenesis of narcolepsy. Genetic susceptibility (top) is dominated by the HLA-DQB1*06:02 allele within the major histocompatibility complex on chromosome 6, which is present in the vast majority of individuals with narcolepsy type 1 and confers a strong, but not deterministic, predisposition. Additional HLA and non-HLA loci contribute modestly, supporting a polygenic model of immune vulnerability. Autoimmune mechanisms (upper middle) are postulated to arise from antigenic mimicry following infections such as influenza or streptococcal illness, leading to activation of autoreactive CD4^+^ and CD8^+^ T cells that selectively target orexin-expressing neurons in the lateral hypothalamus. H1N1 hemagglutinin has been implicated as a molecular trigger in this process. Cell silencing and neuronal vulnerability (lower middle) suggest that surviving orexin neurons may remain anatomically present but transcriptionally inactive, potentially through epigenetic suppression of the orexin gene. Experimental observations indicate that certain neuromodulators (e.g., morphine) can upregulate orexin expression, raising the possibility of latent neuronal rescue. Epigenetic and additional factors (bottom), including altered DNA methylation and related regulatory mechanisms, may toggle orexin expression between functional and silent states, thereby shaping phenotypic variability. Although autoantibodies against orexin neurons have been proposed, consistent detection in humans remains lacking. Together, this multi-hit model integrates genetic risk, immune activation, and epigenetic modulation as convergent drivers of orexin neuron dysfunction or loss.

Together, these findings support a model in which genetic and epigenetic vulnerabilities lead to immune system dysfunction; environmental triggers, such as infections, initiate inflammatory responses; and T cell–mediated damage or silencing of orexin neurons trigger sleep-wake control to fail (**Figure 1**). This framework explains the differences across narcolepsy types, accounts for mismatches between orexin levels and clinical features, and positions NT1 as a typical autoimmune encephalopathy (46, 47). This reconceptualization increasingly motivates investigations of early immunomodulatory therapies. In parallel, proof-of-concept studies exploring orexin receptor–targeted pharmacology and cell replacement are gaining traction as restorative strategies (12, 49).

However, significant gaps remain: definitive human evidence of antigen-specific cytotoxicity remains elusive, early biomarkers of immune activation have not yet been validated, and *in vivo* imaging capable of detecting hypothalamic inflammation is still unavailable. Nonetheless, recent mechanistic advances have refined a biologically coherent model of disease pathogenesis. The key challenge now is to translate these insights into interventions that can modify the disease’s trajectory and, ultimately, prevent or reverse orexin neuronal failure.

## 5 Pathological findings

Neuropathological investigations consistently demonstrate that NT1 is characterized by a striking, highly selective loss of orexin-producing neurons in the lateral hypothalamus. Quantitative stereological studies estimate that approximately 75–95% of the ~50,000–70,000 orexin neurons are absent in most NT1 brains, while neighboring neuronal populations remain remarkably preserved (46, 47). Adjacent melanin-concentrating hormone neurons are spared, and inflammatory hallmarks are minimal—typically limited to subtle gliosis rather than frank lymphocytic infiltration or degenerative pathology (46, 50).

A central unresolved question is whether the missing neurons are irreversibly destroyed or survive in a transcriptionally silent state, masked by suppression of orexin gene expression (51).

Experimental models have long demonstrated a dose–response relationship between the extent of orexin neuron loss and clinical disease severity: partial depletion produces REM intrusion and sleep-wake fragmentation but may spare cataplexy, often with preserved CSF orexin levels (46). Human postmortem studies reinforce this continuum. In NT2, orexin neuron counts may be normal or modestly reduced (approximately 30–35%) with relative sparing of the posterior hypothalamic regions, supporting a continuum of selective vulnerability rather than a binary presence or absence of neuronal loss (47, 52).

Alongside orexin depletion, recent studies report an apparent increase in histaminergic neurons in the tuberomammillary nucleus (TMN) of NT1 brains. Postmortem studies using histidine decarboxylase (HDC) immunohistochemistry have reported a higher number of HDC-immunoreactive TMN neurons than controls; however, reported magnitudes vary and reflect differences in methodology, including whether unbiased stereological counting or marker-based cell identification was used (47, 52). Neuroimaging studies have echoed these findings, suggesting regionally altered hypothalamic structure or signal characteristics *in vivo*, although current imaging modalities do not directly quantify histaminergic neuron number and should be interpreted cautiously (50). These findings are commonly interpreted as compensatory plasticity within a wake-promoting system—emphasizing the reciprocal connectivity between TMN histamine neurons and orexin circuits.

However, substantial methodological uncertainty persists. Differences in immunolabeling protocols, fixation artifacts, altered expression of histidine decarboxylase, or recruitment of neurons previously below the threshold for histaminergic markers could inflate apparent neuron counts (47, 52). Importantly, an increase in HDC-immunoreactive profiles does not necessarily equate to neurogenesis, as enhanced enzyme expression or phenotypic plasticity may render previously low-expressing cells detectable. Whether NT1 brains truly gain new histaminergic neurons, reprogram existing cells to adopt histaminergic identity, or merely upregulate marker expression remains unresolved. The paradox of profound orexin neuron depletion in the absence of overt neurodegeneration supports a mechanism of selective immune-mediated vulnerability rather than global hypothalamic injury (46, 51).

Taken together, the neuropathological evidence supports a coherent model: NT1 is characterized by near-complete, spatially restricted loss of orexin neurons, with preservation of surrounding hypothalamic architecture. The proposed amplification of histaminergic neurons represents a provocative yet incompletely validated adaptive response—one that demands deeper, multimodal evaluation integrating modern stereology, molecular markers, and functional imaging.

## 6 Genetic and epigenetic factors

Narcolepsy presents a paradoxical genetic architecture: it is predominantly sporadic in clinical practice, yet it carries some of the strongest immunogenetic associations in sleep medicine. Familial aggregation is uncommon. Twin studies estimate concordance of roughly 20–30% among monozygotic pairs, reinforcing the notion that genetic predisposition is necessary but insufficient to produce the phenotype (36, 43). Large pedigrees with multiple affected individuals remain exceptional, and familial forms constitute only a small minority of cases worldwide (53). These observations highlight the primacy of non-genetic triggers, particularly immune and environmental events, in shaping disease onset.

The most robust genetic association continues to arise from the HLA complex. HLA-DQB1*06:02 is present in approximately 85–98% of individuals with NT1 and in a substantial subset of those with NT2 (46, 47). Nevertheless, its low penetrance—estimated at roughly 1 in 1,000 carriers—underscores its permissive rather than deterministic role. Intriguingly, the allele is common in healthy individuals, and its presence correlates with shorter REM latency even in asymptomatic carriers, suggesting a subclinical signature of altered arousal regulation (36).

Population-level variation in allele frequency appears to influence disease incidence: for example, reductions in DQB1*06:02 prevalence in certain Middle Eastern and Jewish populations parallel their lower rates of narcolepsy (36). Other HLA class II alleles show more modest, context-specific associations, whereas emerging evidence suggests that class I loci influence susceptibility through CD8^+^ T-cell-mediated mechanisms (46).

Genetic studies beyond the HLA region continue to converge on T-cell signaling and antigen-recognition pathways. Polymorphisms in the T-cell receptor α locus replicate across independent cohorts and reinforce the importance of antigen-specific immune response presentation in disease susceptibility (36). Additional loci, including *P2RY11*, *CTSH*, *TNFSF4*, *IFNAR1*, *ZNF365*, *DENND1B*, *IKZF4–ERBB3*, *SIRPG*, *CD207* and *PRF1*—have been identified in genome-wide association studies, with effect sizes characteristic of a polygenic autoimmune phenotype (46, 53). Although rare mutations in *HCRT*, *MOG*, or *P2RY11* occasionally occur in familial cases, none reliably reproduce the full-spectrum clinical phenotype in large series (53).

Epigenetic modulation has emerged over the past decade as a compelling additional layer of vulnerability. Epigenome-wide association studies suggested global alterations in DNA methylation patterns, with enrichment in pathways related to hormonal regulation and cellular metabolism (36). More provocative findings arise from postmortem analyses showing promoter hypermethylation of the *HCRT* gene, decreased chromatin accessibility, and preserved expression of adjacent neuropeptides in orexin-lineage neurons—raising the possibility that some orexin neurons may remain anatomically present but epigenetically silenced rather than destroyed (54).

Reviews published between 2023 and 2025 increasingly emphasize the potential role of DNA methylation, histone modification, and noncoding RNA dynamics in shaping immune tolerance and neuronal vulnerability (44, 46).

Recent multi-omics integration studies further refine our understanding of risk. Different studies link specific single-nucleotide polymorphism clusters to sleep efficiency, microarousal dynamics, and REM instability, suggesting that genetic background not only shapes disease susceptibility but also modulates clinical phenotype expression and disease course (36, 55, 56). These observations support a future in which polygenic and epigenetic risk profiles may guide risk stratification, enable identification of preclinical individuals, predict trajectories of symptom evolution, and guide precision-oriented interventions.

In synthesis, the genetic and epigenetic architecture of narcolepsy reflects a highly penetrant immunogenetic core centered on HLA and TCR pathways, which is modulated by numerous secondary immune-regulatory loci. Epigenetic regulation provides a flexible, potentially reversible layer that can amplify or reduce vulnerability. There is growing evidence suggesting that environmental triggers interact with these molecular frameworks to precipitate the disease. To advance the field, there is an urgent need for deep profiling of methylomic and transcriptomic signatures in at-risk populations, single-cell epigenetic analysis of orexin-lineage neurons and immune cells, and the development of integrative multi-omics risk models based on prospective cohorts.

## 7 Environmental factors

The modest concordance observed in monozygotic twins—typically around 20–30%—strongly suggests that environmental modifiers exert substantial influence over narcolepsy pathogenesis (1, 36). Epidemiological studies suggest that both prenatal and early-life exposures shape susceptibility. Season-of-birth effects, although inconsistent across cohorts, suggest that early encounters with viral infections, immune-priming events, or inflammatory stressors may influence the developing immune system and modulate the long-term risk of autoimmunity (53, 57).

Historical accounts from the early 20th century documented temporal clustering of narcolepsy onset around outbreaks of influenza and post-encephalitic syndromes, raising the possibility that strong immune activation can precipitate disease in genetically primed individuals (1). More recent serological studies provide convergent evidence: individuals with new-onset narcolepsy exhibit elevated anti–streptococcal antibody titers relative to matched controls, supporting the hypothesis that group A β-hemolytic streptococcal infection may trigger narcolepsy in susceptible hosts (58).

Among infectious exposures, influenza A/H1N1 stands out as the most consistent environmental risk factor. During the 2009–2010 pandemic, incidence of narcolepsy in China increased approximately threefold shortly after peak infection periods; in northern European countries, a dramatic six- to ninefold rise occurred following administration of the AS03-adjuvanted Pandemrix vaccine, an effect not observed with other influenza vaccines (57, 59). These observations underscore that both natural infection and, in rare circumstances, vaccine-associated immune activation may breach immune tolerance mechanisms.

Mechanistic hypotheses center on molecular mimicry. Experimental work demonstrates sequence homology between influenza hemagglutinin epitopes and orexin-related peptides, suggesting that cross-reactive T cells could inadvertently target orexin neurons during antiviral immune responses (3, 5). Sporadic case reports also describe narcolepsy onset following other immunogenic events—including routine vaccinations or traumatic brain injury—further supporting a multi-hit architecture in which strong inflammatory stimuli act as proximal triggers (53).

More recently, isolated but well-documented cases have linked SARS-CoV-2 infection with *de novo* narcolepsy in genetically predisposed individuals. A 2023 case report described a patient with HLA-DQB1*06:02 who developed abrupt-onset hypersomnolence and cataplexy following COVID-19, accompanied by low CSF orexin levels, reinforcing the plausibility of virus-induced immune dysregulation (60).

Taken together, environmental modifiers appear to act across distinct temporal stages. Early-life exposures may shape immune setpoints, influence synaptic pruning and regulate T-cell tolerance. In contrast, later exposures—such as respiratory infections, streptococcal immune activation or pandemic viral pathogens—may serve as decisive triggers that push a primed immune system toward autoreactivity. These patterns align with a modern multi-hit framework in which genetic predisposition, epigenetic tuning and immune stressors converge to breach tolerance and initiate selective loss or silencing of orexin neurons.

## 8 Immunological mechanisms

Despite decades of investigation, narcolepsy has not been associated with a disease-specific autoantibody. Unlike classical antibody-mediated autoimmune encephalitides, narcolepsy shows no reproducible humoral biomarkers. However, epidemiological patterns, immune associations and therapeutic observations collectively point toward an immune-mediated pathophysiology predominantly involving adaptive cellular immunity.

Narcolepsy has been described in association with systemic autoimmune disorders—including multiple sclerosis, coeliac disease and systemic lupus erythematosus—as well as in rare paraneoplastic contexts. These associations suggest that global immune dysregulation may create a biological milieu permissive to selective hypothalamic vulnerability rather than reflecting shared antigenic targets (46, 53). Reports of early benefit from immunomodulatory therapy in selected patients, although inconsistent, further reinforce the potential reversibility of early immune-driven processes (51). Notably, the absence of a consistent autoantibody distinguishes narcolepsy from antibody-driven limbic or diffuse autoimmune encephalitides and aligns it more closely with T-cell-mediated neurological disorders.

Ancillary CSF findings offer partial but suggestive support for immune activation. Some patients exhibit mild pleocytosis, oligoclonal bands, or elevated cytokine levels, such as TNF-α and IFN-γ, though these abnormalities lack sensitivity and are inconsistently observed across cohorts (46). Flow-cytometric and functional immune studies demonstrate enhanced activation of both CD4^+^ and CD8^+^ T cells in peripheral blood, with parallel activation patterns observed in CSF in subsets of patients (45).

A pivotal advance occurred in 2018, when investigators first identified autoreactive CD4^+^ and CD8^+^ T cells that recognize orexin-related epitopes in individuals with NT1 and NT2 (3). These findings were independently validated in subsequent studies that confirmed T-cell receptor specificity for orexin and structurally related peptides (4, 5). Together, these studies provide strong mechanistic support for the involvement of antigen-directed T cells in the selective targeting of orexin-producing neurons in narcolepsy, rather than indicating a nonspecific neuroinflammatory process.

Recent work has expanded this immunological framework. Transcriptomic profiling of circulating T cells in narcolepsy reveals altered expression signatures enriched for T-cell receptor signaling, immune activation pathways, and effector differentiation, suggesting sustained systemic dysregulation (45). Mendelian randomization studies implicate traits of both CD4^+^ and CD8^+^ T-cell activation as causally related to NT1, thereby linking immunogenetic susceptibility directly to functional immune phenotypes (61).

At the tissue level, a 2025 postmortem study reported an approximately eleven-fold increase in CD4^+^ T-cell density within the orexin neuronal field, far exceeding levels observed in adjacent hypothalamic regions and control brains, supporting anatomically restricted immune infiltration rather than diffuse hypothalamic inflammation (46, 51).

Despite these advances, the precise effector mechanisms underlying orexin neuron dysfunction remain incompletely defined; however, accumulating evidence is increasingly consistent with narcolepsy—particularly NT1—falling within the spectrum of T cell–mediated autoimmune neurological disorders. The absence of a consistent humoral biomarker reinforces the view that narcolepsy aligns more closely with cellular autoimmune diseases than with antibody-mediated encephalitides. Recognizing narcolepsy as an immune-mediated encephalopathy has important clinical implications: it highlights the urgent need for reliable biomarkers of early immune activation, motivates trials of immunomodulatory interventions in the earliest phases of illness, and challenges the long-standing assumption that orexin neuronal loss is irreversible.

Microglial activation has emerged as a potential amplifier of immune-mediated vulnerability. Experimental models demonstrate that orexin deficiency itself enhances microglial reactivity and inflammatory signaling, suggesting a bidirectional relationship in which loss of orexin function both results from and contributes to neuroimmune dysregulation (46). Whether microglia act as primary effectors or secondary responders in human narcolepsy remains unresolved.

In summary, available evidence positions narcolepsy, particularly NT1, within the spectrum of T cell–mediated autoimmune neurological disorders, albeit with distinctive features: profound cellular selectivity, minimal structural inflammation, and absence of humoral biomarkers. This profile contrasts sharply with classical autoimmune encephalitides and supports the conceptualization of narcolepsy as an immune-mediated hypothalamic encephalopathy rather than an antibody-driven inflammatory brain disease.

Future research must therefore prioritize precise immunophenotyping, longitudinal immune monitoring from prodromal stages, and mechanistic studies capable of disentangling immune-driven neuronal silencing from irreversible cell loss. Such efforts will be essential to determine whether narcolepsy represents a preventable or partially reversible immune-mediated disorder—and to translate immunological insight into effective disease-modifying therapies.

## 9 Clinical features

**Figure 2:**
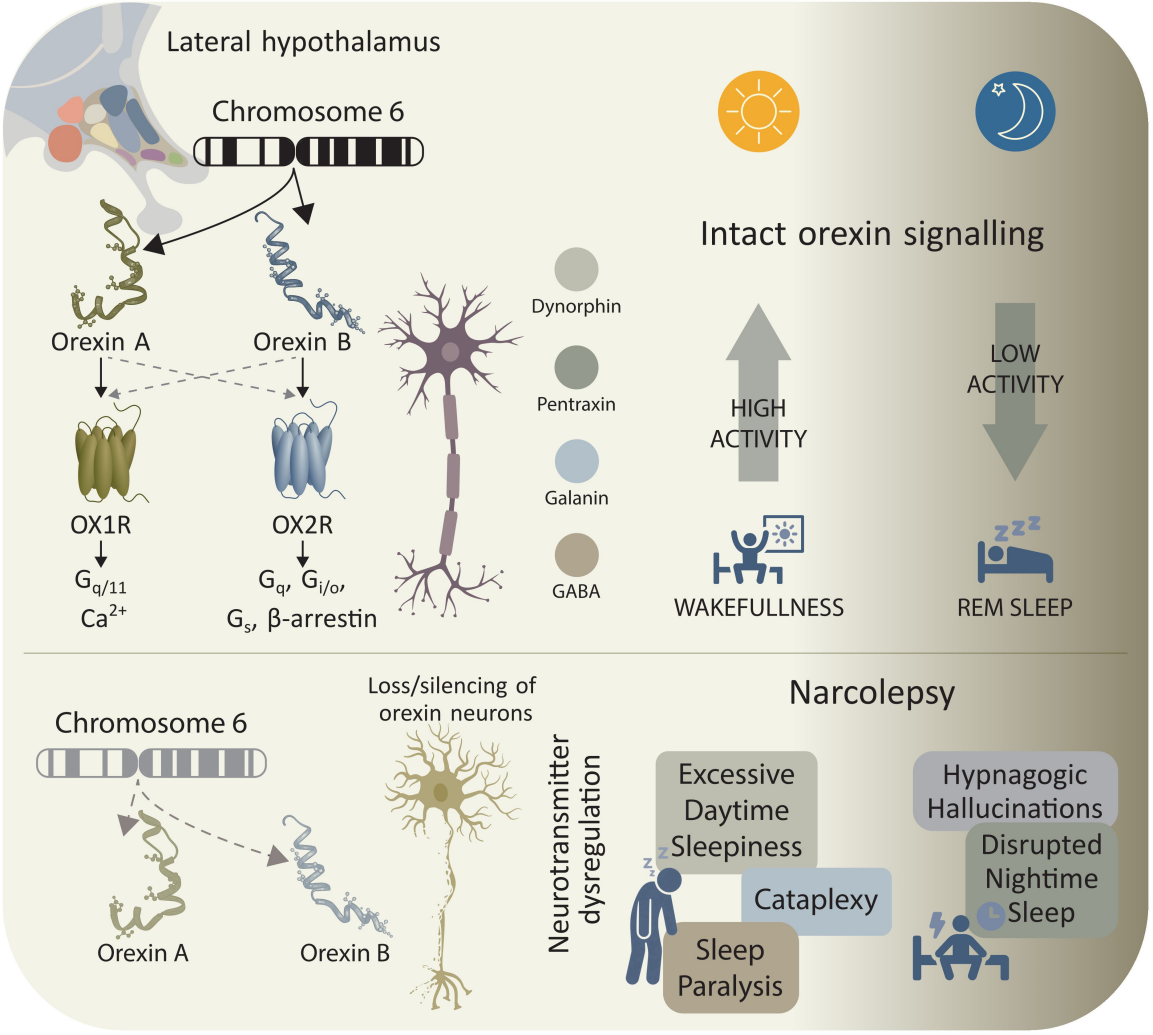
Orexin neuron dysfunction and state instability in narcolepsy. In physiological conditions (upper panel), orexin-A and orexin-B—encoded on chromosome 6—are released by approximately 70,000 neurons in the lateral hypothalamus. These peptides activate orexin receptors OX_1_R and OX_2_R across histaminergic, monoaminergic, and cholinergic arousal networks to maintain consolidated wakefulness. OX_1_R primarily couples to G_q/11_-mediated intracellular Ca²^+^ signaling, whereas OX_2_R engages G_q_, G_i/o_, and G_s_ pathways and β-arrestin signaling. Orexin neurons co-release excitatory neuromodulators, including dynorphin, neuronal pentraxin, galanin, and GABA, providing tonic excitation that prevents inappropriate REM sleep intrusions. In narcolepsy (lower panel), immune-mediated loss or silencing of orexin neurons abolishes this stabilizing drive, leading to excessive daytime sleepiness, sudden emotion-triggered loss of muscle tone (cataplexy), and transitions into REM-associated paralysis while awake (sleep paralysis). Dysregulated neurotransmitter signaling leads to rapid, uncontrolled shifts between behavioral states. During normal wakefulness, orexin neuron activity is high; during sleep, activity falls. In orexin deficiency, these boundaries become unstable. REM sleep features—including atonia and dream-related hallucinations—intrude into wakefulness, while nighttime sleep becomes fragmented with frequent arousals, contributing to hypnagogic hallucinations, poor nocturnal sleep quality, and persistent daytime impairment. Overall, disrupted orexin signaling produces pathological state instability that defines the narcolepsy phenotype.

Narcolepsy type 1—and to a lesser extent narcolepsy type 2—should be understood not merely as a disorder of sleep-wake instability, but as a multisystem syndrome rooted in hypothalamic dysfunction, with manifestations spanning motor, psychiatric, cognitive, metabolic and autonomic domains (**Figure 2**).

### 9.1 Excessive daytime sleepiness

Excessive daytime sleepiness remains the hallmark and most disabling symptom. Patients describe an overwhelming sleep drive or persistent drowsiness, impaired vigilance, difficulty sustaining wakefulness, and episodes of irresistible sleep attacks (11, 34, 39). In a large European cohort of over 1,000 patients, approximately 80% reported involuntary napping—typically abrupt, often occurring in the morning—and occasionally in unsafe contexts (62). These naps are often brief (~15–20 minutes), restorative in some cases, and may include dreamlike features; yet their duration and restorative quality vary widely. “Automatic behaviors”—the unconscious continuation of task performance during microsleeps (for example, typing, driving, misplacing objects)—are common, often accompanied by amnesia and experienced by patients as “black-outs” (34, 39, 62). Notably, EDS must be distinguished from fatigue: up to 60% of patients report persistent fatigue, which tends to resist conventional therapy and adds an independent layer of functional impairment (34, 39, 62).

### 9.2 Cataplexy

Cataplexy is the only pathognomonic feature of narcolepsy: brief, transient, emotion-triggered muscular atonia with preserved consciousness. Partial attacks lasting 2–10 seconds frequently involve facial droop, eyelid closure, jaw sagging, tongue protrusion, or extremity weakness; the so-called “facies cataplectica” (especially in pediatric-onset cases) is characterized by mouth opening, facial hypotonia, and tongue protrusion (39, 63). Deep-tendon reflexes typically attenuate or disappear during full cataplexy, although milder attacks may preserve residual reflexes. Rare phenomena, such as a transient Babinski sign or the persistence of a Parkinsonian tremor, have been described, though these remain exceptional (39, 63). Cataplectic episodes generally last less than 2 minutes; durations of more than 5 minutes are uncommon and often reflect withdrawal from anticataplectic therapy (39, 63). Some attacks manifest mixed motor signs: positive motor phenomena (twitching, grimacing, neck extension) may accompany underlying atonia, particularly in children, and occasionally mimic focal convulsive or movement disorders (39, 63). Predominant triggers are positive emotions (laughter being the classic example), with up to half of patients reporting attacks triggered by tickling. Unexpected or triumphant emotions (sports, games, erotic stimuli) may trigger generalized atonia (“orgasmolepsy”). Negative emotions (anger, fear, sorrow) trigger attacks less commonly. During cataplexy, ocular motility and respiration are typically preserved, but some patients report blurred vision, dyspnea or autonomic signs, including fluctuations in blood pressure, sweating, penile erection or urinary incontinence (39, 63). Prolonged cataplexy may be accompanied by concurrent hypnagogic hallucinations, sleep paralysis, vivid dreaming or REM behavior elements (39, 63).

### 9.3 Sleep paralysis and hallucinations

Sleep paralysis and hallucinations occur in approximately 50–60% of patients (34, 39, 63). Sleep paralysis is characterized by a transient inability to move or speak during transitions into or out of sleep, often accompanied by respiratory discomfort. Hallucinations are vivid, multimodal (visual, auditory, olfactory, gustatory, vestibular), and frequently blend seamlessly into the patient’s immediate surroundings. Purely isolated visual hallucinations are less common (~15%) (64). Patients sometimes report a sensed presence, intrusive agents or assault scenarios, which can provoke fear of sleep and anxiety; most maintain insight upon awakening, but the experience may confound psychiatric diagnoses. Rarely, dream enactment (“dream delusions”) occurs beyond the typical hypnagogic spectrum (34, 39, 63).

### 9.4 Sleep disturbances and parasomnias

Although EDS dominates the daytime picture, nocturnal sleep in narcolepsy is often fragmented, with recurrent awakenings and micro-arousals; total sleep time does not necessarily exceed that of the general population (34, 39, 63). In early disease or pediatric cases, prolonged sleep inertia (“sleep drunkenness”), long sleep periods, or paradoxical hypersomnia may be observed (26). Parasomnias are common: periodic limb movements occur in 25–50% of cases, spanning non-REM, REM and wake states, and correlate with both EDS severity and orexin deficiency. REM sleep behavior disorder (RBD) appears in 25–70% of patients, typically manifesting simple behaviors, often paralleling cataplexy burden and orexin loss (34, 39, 63). Other parasomnias—including sleepwalking, nocturnal eating and restless-legs syndrome—are more frequent in narcolepsy than in the general population. Sleep-disordered breathing (e.g., obstructive sleep apnea) is also prevalent and contributes to diagnostic delay (65). Dream content in narcolepsy is often vivid, archaic or bizarre; nightmares, lucid dreaming and dream fragmentation occur more frequently than in the general population (65).

### 9.5 Psychiatric and emotional disturbances

Historically misclassified as psychiatric, narcolepsy nonetheless retains substantive intersections with mood and affective pathology. Stressful life events may precede disease onset, and depression or anxiety affects approximately 20–30% of patients (64). Narcoleptic-like symptoms have also been described in schizophrenia and other psychiatric disorders, and functional mimicry (pseudocataplexy, conversion phenomena) further complicates clinical assessment (34). Neurobiological and animal-model studies implicate dysfunction of reward, limbic and emotional circuits in the genesis of psychiatric symptoms in narcolepsy (66). Psychiatric morbidity is more pronounced in pediatric onset, contributing to learning impairment, social difficulties, low self-esteem and quality-of-life losses comparable to epilepsy (67).

### 9.6 Cognitive disturbances

Cognitive impairment is frequently documented in narcolepsy, including deficits in attention, executive control, processing speed, decision-making and memory (67). Historically, these deficits were primarily attributed to sleepiness, but emerging data suggest that orexin deficiency may directly contribute to impaired protein clearance, neurotoxic accumulation, or synaptic vulnerability (48).

### 9.7 Metabolic disturbances

Elevated body mass index (BMI) and obesity have long been associated with narcolepsy. Contemporary reviews and empirical studies confirm that BMI is 10–20% higher in patients than in matched controls (34). Although insulin sensitivity is often preserved, rates of type 2 diabetes appear increased—likely as a consequence of obesity and metabolic dysregulation (64). Patients exhibit a reduced resting metabolic rate or altered substrate utilization: in one case–control study, narcolepsy patients showed lower respiratory quotients—indicating increased reliance on fat metabolism during fasting—although the resting metabolic rate itself was not significantly different from BMI-matched controls (68). A 2022 narrative review emphasized that orexin deficiency may impair basal metabolic rate, motor activity and energy expenditure, contributing to weight gain over time (68). More recently, a metabolic profiling study in NT1 identified inhibited carbohydrate metabolism and early indicators of diabetic progression (69).

### 9.8 Autonomic dysfunction

Although less studied, autonomic disturbances are increasingly documented. Patients may report syncope or presyncope, erectile dysfunction, night sweats, gastrointestinal dysmotility, orthostatic hypotension, palpitations, dry mouth, thermoregulatory instability or pupillary irregularities (34). Olfactory dysfunction, chronic headache and back pain have also emerged as associated features, though their mechanistic linkage to narcolepsy remains uncertain (65).

### 9.9 Pediatric presentation

In children, early clues often include rapid weight gain and onset of EDS; paradoxically, despite pronounced daytime sleepiness, children may maintain long nocturnal sleep durations. This combination of excessive daytime sleepiness with preserved or prolonged nocturnal sleep has been consistently described in pediatric cohorts and may delay recognition when interpreted as “normal sleep need” (27, 70). Attempts to resist EDS may manifest as restlessness or hyperactivity. Such paradoxical hyperactivity and attentional dysregulation are frequently misattributed to primary ADHD rather than hypersomnolence (27).

Cataplexy typically emerges early and frequently presents with combined negative motor signs (facial atonia, ptosis, mouth opening, tongue protrusion) and positive motor phenomena (dystonia, dyskinesia, stereotypies)—particularly in the first year of disease onset—and tends to diminish over time (67). Primary pediatric studies further describe “cataplectic facies,” complex motor instability and frequent non–emotion-triggered hypotonic episodes in early-onset cases, especially in preschool-aged children (67, 70). Longitudinal cohort data suggest that this complex motor phenotype often evolves toward more classic emotion-triggered cataplexy over time (71).

Children also frequently manifest fragmented sleep, hallucinations, sleep paralysis or RBD, and comorbid cognitive, behavioral and psychiatric symptoms (e.g., depression, attention-deficit/hyperactivity disorder, aggression, psychotic features) are common (67). Multiple pediatric reviews confirm high rates of mood disorders, anxiety, attentional dysfunction and school impairment, although prevalence estimates vary across cohorts and are largely based on cross-sectional data (27, 67). Moreover, metabolic and neuroendocrine features—such as obesity and early puberty—are more prominent in pediatric onset, underscoring early hypothalamic involvement (67). Rapid weight gain at disease onset is well documented in pediatric NT1 cohorts and is considered an evidence-based clinical marker of early disease, whereas mechanistic attribution to hypothalamic neuroendocrine disruption remains inferential (70, 71).

Over the past five years, the clinical landscape of narcolepsy has been refined through patient-centric surveys, metabolic phenotyping and advances in neuroimaging (e.g., advanced MRI biomarkers of hypothalamic integrity) (65). Nevertheless, key challenges remain: disentangling the direct contributions of orexin deficiency from secondary sleep loss, identifying early prodromal biomarkers and capturing the full spectrum of systemic effects in longitudinal studies.

## 10 Diagnosis

**Figure 3:**
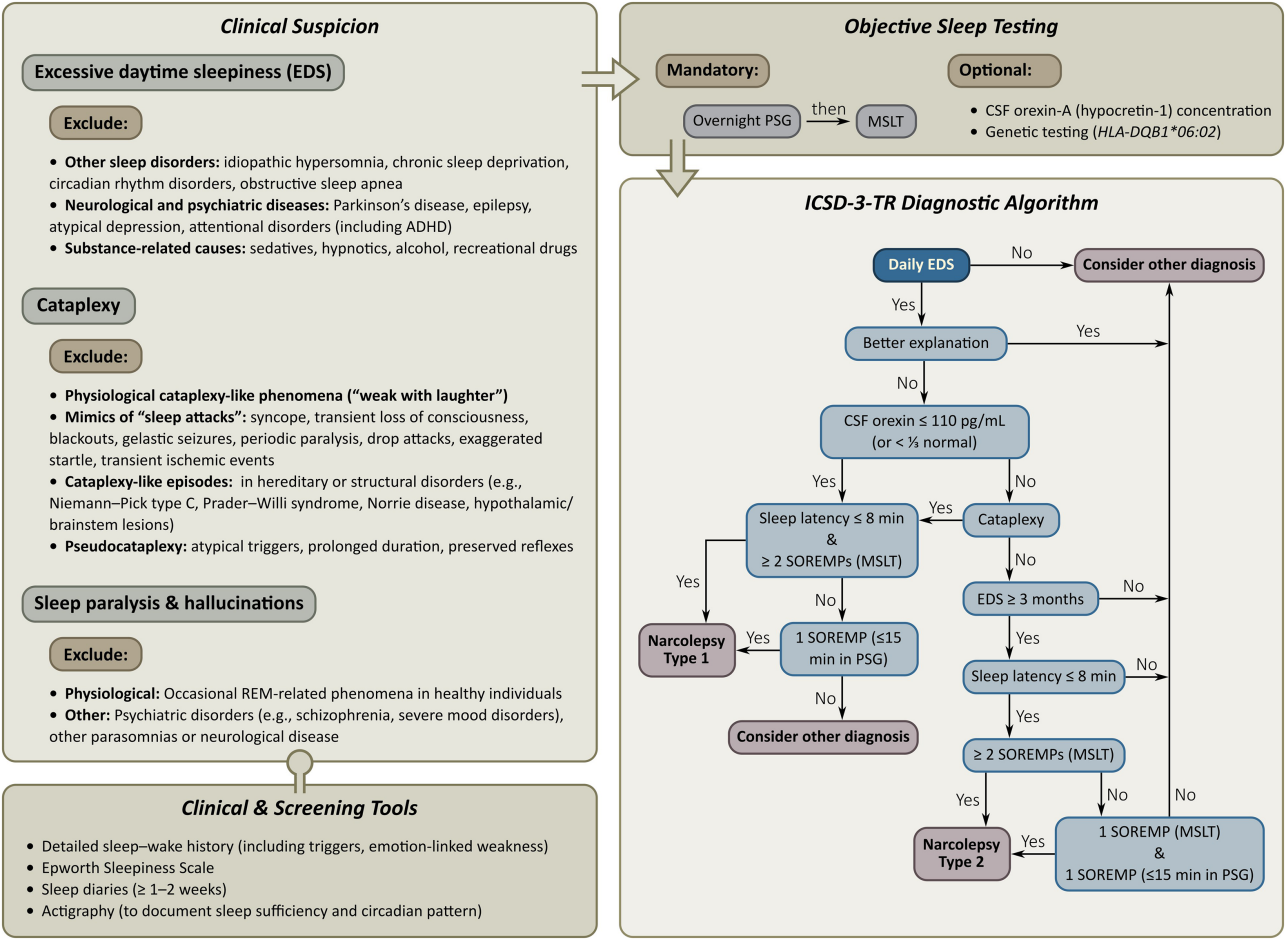
Diagnostic approach for narcolepsy according to current ICSD-3-TR criteria. Diagnosis begins with clinical suspicion based on the cardinal symptoms of narcolepsy—EDS, cataplexy, sleep paralysis, and hallucinations—followed by systematic exclusion of alternative conditions that better explain these manifestations. Differential diagnoses include other sleep disorders, neurological and psychiatric diseases, substance-related causes, and physiological or pathological mimics, particularly for cataplexy and REM-related phenomena. Clinical and screening tools (detailed sleep-wake history, Epworth Sleepiness Scale, sleep diaries, and actigraphy) may support phenotyping and exclusion of confounders. Objective sleep testing with overnight PSG followed by MSLT is mandatory, whereas CSF orexin-A (hypocretin-1) measurement and genetic testing (HLA-DQB1*06:02) are optional ancillary investigations. Final classification as narcolepsy type 1 or type 2 is based on application of ICSD-3-TR diagnostic criteria, integrating clinical features, exclusion of alternative explanations, and objective findings. EDS, excessive daytime sleepiness; PSG, polysomnography; CSF, cerebrospinal fluid; MSLT, multiple sleep latency testing; SOREMP, sleep-onset REM periods.

Narcolepsy remains substantially underdiagnosed, with diagnostic delays that continue to impede timely management. In the largest contemporary European cohort, the average interval between symptom onset and correct diagnosis approached 14 years, reflecting persistent gaps in clinical recognition and structural barriers within sleep-medicine pathways (29, 62). These delays are particularly pronounced in children, whose early manifestations of excessive daytime sleepiness and cataplexy may be subtle, atypical or misinterpreted. Normative data for the multiple sleep latency test (MSLT) remain limited in younger children, and CSF orexin measurement—especially in those under age six—remains infrequently performed due to procedural and ethical constraints (34). **Figure 3** outlines the diagnostic approach for narcolepsy according to current ICSD-3-TR criteria (9).

### 10.1 Narcolepsy type 1

Current classifications define narcolepsy type 1 by persistent EDS for at least three months, together with either unequivocal CSF orexin deficiency (≤ 110 pg/mL using non-standardized assays) or the presence of cataplexy plus characteristic polysomnographic criteria—mean sleep latency < 8 minutes and ≥ 2 sleep-onset REM periods (SOREMPs) on the MSLT (8). In practice, many diagnoses rely primarily on clinical history, as cataplexy remains pathognomonic and can be recognized with careful, structured interviewing. Nevertheless, reliable documentation of cataplexy remains challenging: validated trigger tests are limited and attempts to capture episodes via video or electrophysiological recordings have yielded only modest gains in diagnostic confidence (72).

Even in seemingly straightforward NT1 cases, diagnostic uncertainty persists. Immunoassays for CSF orexin show considerable methodological variability; cross-reactivity, peptide degradation and lack of interlaboratory standardization continue to undermine reliability (73). A stability study demonstrated notable variability in stored CSF samples, underscoring the need for caution when interpreting orexin concentrations near diagnostic thresholds (73). Moreover, intermediate orexin levels—neither clearly low nor convincingly normal—pose a diagnostic challenge, often requiring longitudinal reassessment or probabilistic modelling to contextualize risk (42).

### 10.2 Narcolepsy type 2

Persistent EDS defines narcolepsy type 2 in the absence of cataplexy, a mean MSLT latency < 8 minutes, ≤ 2 SOREMPs, and normal (or unmeasured) CSF orexin. NT2 remains a diagnosis of exclusion and therefore demands rigorous evaluation for contributory conditions such as insufficient sleep, obstructive sleep apnea, circadian misalignment, sedative medications and psychiatric disorders. Although NT2 patients typically exhibit milder EDS and fewer REM-intrusion symptoms than NT1 patients, the entity remains controversial. Evidence that some NT2 patients progress to NT1, that a subset demonstrates partial orexin neuron loss on postmortem examination, and that partial orexin depletion in animal models faithfully reproduces EDS without cataplexy all support the concept of a disease continuum rather than a categorical distinction (47, 74).

### 10.3 Limitations of current diagnostic criteria

The limitations of existing diagnostic frameworks have become increasingly apparent. First, polysomnographic metrics such as SOREMPs and MSLT latency are vulnerable to confounding by insufficient sleep, comorbid sleep disorders, medication effects and suboptimal testing conditions, reducing their specificity (75).

Second, although orexin deficiency is a powerful biomarker, current immunoassays lack standardization; cross-reactive metabolites and peptide breakdown products may contribute to false-low or ambiguous results (42, 73). CSF orexin-A quantification has historically relied on radioimmunoassay (RIA), from which the widely adopted diagnostic threshold of <110 pg/mL (or <1/3 of mean control values) was originally derived. However, newer analytical platforms yield systematically different absolute concentrations. ELISA-based methods have reported values approximately fourfold lower than RIA in identical samples (76), and liquid chromatography–tandem mass spectrometry (LC–MS/MS), which selectively quantifies intact mature orexin-A, has demonstrated concentrations three- to fivefold lower than RIA (73). These discrepancies likely reflect differences in antibody specificity, epitope recognition and calibration strategies, underscoring that absolute cut-offs are assay-dependent and not directly interchangeable across laboratories. Moreover, pre-analytical factors—including sample handling, storage temperature, duration of storage and repeated freeze–thaw cycles—affect peptide stability and may further influence measured concentrations (73), emphasizing the need for standardized protocols.

Third, operational criteria for cataplexy remain imprecise. The absence of quantitative thresholds, reproducible elicitation protocols and validated rating tools hampers inter-center diagnostic consistency. Recent data indicate that individuals with intermediate CSF orexin-A concentrations (above 110 pg/mL but below conventional laboratory reference ranges) may still exhibit typical cataplexy and fulfill PSG/MSLT criteria for narcolepsy type 1, suggesting that strict dichotomous thresholds may fail to capture clinically meaningful partial deficiency (77). Interpretation of intermediate values, therefore, requires careful clinical correlation rather than reliance on a single numerical cut-off.

Fourth, existing criteria insufficiently capture the clinical heterogeneity of narcolepsy. Intermediate phenotypes—such as individuals with partial orexin deficiency, sporadic SOREMPs or fluctuating symptom clusters—are poorly served by rigid dichotomous classifications. HLA-DQB1*06:02 positivity, SOREMPs and reduced orexin levels have all been reported in individuals without overt narcolepsy, suggesting a broader latent phenotype spanning susceptibility to subclinical REM dysregulation (36, 47).

Given these limitations, a revision of diagnostic nosology is warranted. A more contemporary, spectrum-based model would integrate multidimensional axes: clinical phenotype (symptom clusters, severity, progression), biomarker stratification (CSF, high-resolution imaging, genetic and immunologic markers), and etiological subtype (autoimmune, secondary, latent). Such a framework would better accommodate evolving phenotypes—such as patients transitioning from isolated EDS to NT1—and identify opportunities for earlier intervention (see **Tables 1**, **2**).

### 10.4 Emerging and prospective directions

Recent post-pandemic analyses confirm that narcolepsy remains substantially under-recognized globally (75). This under-recognition is particularly pronounced in pediatric populations, where diagnostic delays of up to a decade have been repeatedly documented, and early-onset cases may present with atypical or evolving phenotypes (27, 67, 70). Advances in molecular and neuroimaging tools offer promising avenues for diagnostic refinement. High-sensitivity liquid chromatography–mass spectrometry (LC–MS) orexin assays may overcome the limitations of current immunoassays, improving accuracy and enabling simultaneous quantification of multiple orexin-related peptides (75). Orexin receptor imaging, CSF proteomics and immune-signature profiling represent emerging approaches that may help define early disease stages and reduce reliance on MSLT-dependent metrics. In pediatric NT1, where rapid weight gain, endocrine dysregulation, and complex motor phenomena may precede classical cataplexy, such biomarker-driven approaches could facilitate earlier pathophysiological stratification (71). However, these techniques remain investigational and are not yet supported by pediatric validation cohorts.

Management of intermediate orexin values increasingly incorporates Bayesian and longitudinal models that integrate repeated sampling, symptom evolution and risk characteristics (42). In children, this longitudinal approach is particularly relevant, as symptom expression may evolve from complex hypotonic facial or generalized motor phenomena to more typical emotion-triggered cataplexy over time (70, 71).

In pediatric populations, investigators are developing age-appropriate MSLT standards, exploring the use of daytime polysomnography, and evaluating actigraphy-supported diagnostic pathways to mitigate confounding effects of sedation and developmental variability (78). Current pediatric diagnostic thresholds (mean sleep latency ≤8 minutes with ≥2 SOREMPs) are extrapolated from adult-based ICSD criteria but are vulnerable to false positives and developmental confounders in children (27, 67). Evidence supporting pediatric-specific MSLT modifications remains limited, largely based on observational and expert consensus data rather than randomized controlled trials. Expert guidelines recommend actigraphy-supported pre-MSLT sleep stabilization protocols and extended sleep logs to exclude insufficient sleep and circadian misalignment, but these recommendations are primarily consensus-based rather than supported by high-level pediatric evidence (27). Similarly, proposals for daytime PSG adaptations or repeat MSLT testing in young children are grounded in clinical experience and cohort data rather than formal pediatric normative trials (70).

Early childhood cases further illustrate diagnostic complexity. Preschool-aged patients may present with facial hypotonia, tongue protrusion, head drop, or frequent non–emotion-triggered cataplectic-like episodes, which are frequently misdiagnosed as epilepsy or movement disorders (67).

In summary, contemporary diagnosis of narcolepsy remains a delicate synthesis of clinical expertise, polysomnography and imperfect biomarkers. In pediatric populations, this synthesis must additionally incorporate developmental context, longitudinal symptom evolution and careful exclusion of behavioral, neurological and sleep-related mimics. To meaningfully reduce diagnostic latency and misclassification, future criteria must integrate biomolecular precision, flexible phenotypic stratification and longitudinal interpretation.

## 11 Pathophysiology

**Figure 4:**
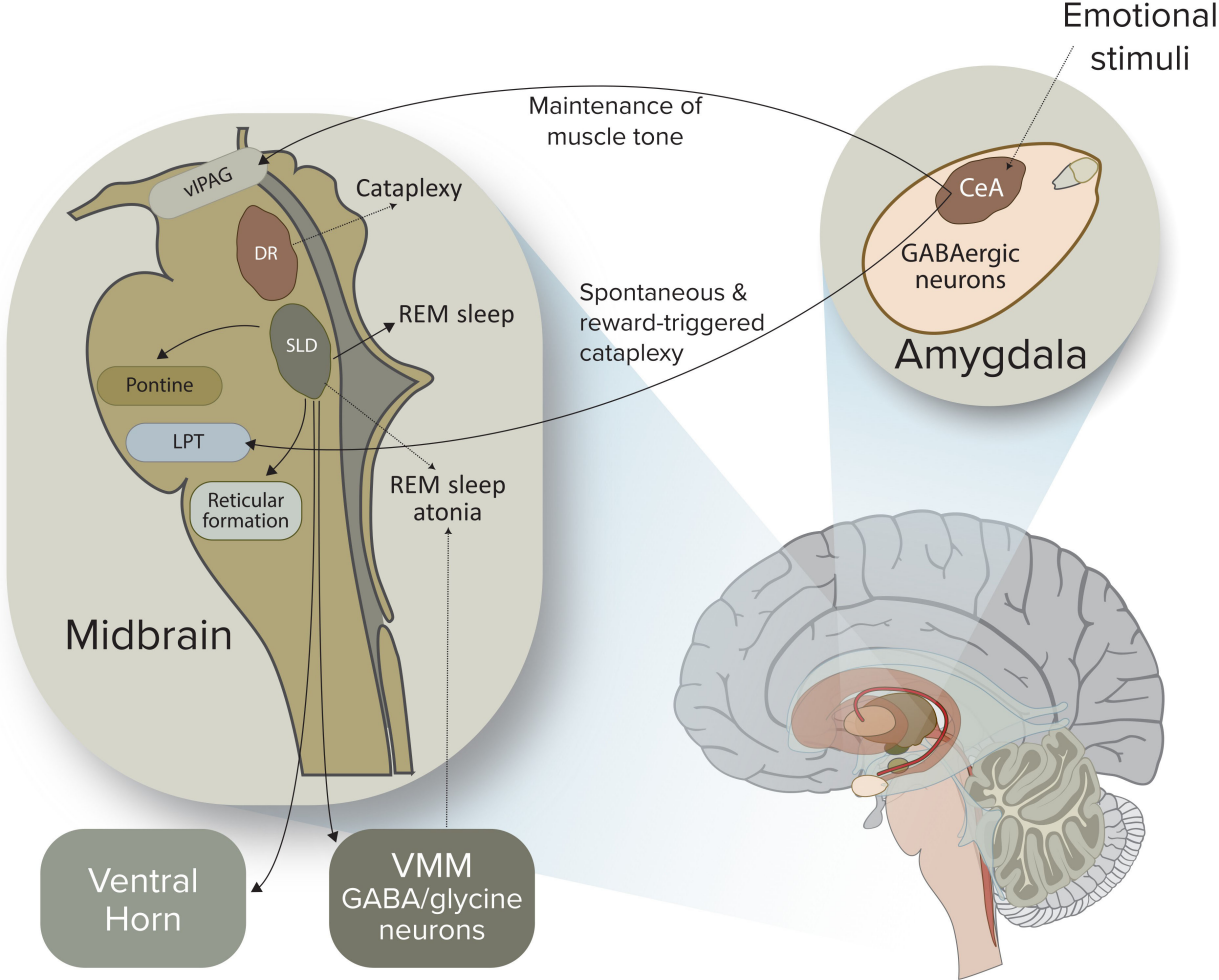
Limbic–Brainstem circuit dysfunction linking emotional triggers to cataplexy and REM-related atonia. Positive emotional stimuli activate GABAergic neurons in the central nucleus of the amygdala (CeA), a key node for reward and affective processing. Under normal conditions, orexin input buffers downstream circuits against inappropriate REM intrusions during wakefulness. In narcolepsy, reduced orexin signaling diminishes excitation of REM-inhibiting structures in the ventrolateral periaqueductal gray (vlPAG), dorsal raphe (DR), and lateral pontine tegmentum (LPT). This imbalance enables amygdala efferents to override the normal gating of REM-sleep circuitry, precipitating sudden and emotionally triggered reductions in muscle tone. When this inhibition fails, the sublaterodorsal nucleus (SLD) becomes aberrantly active, engaging ventromedial medullary (VMM) GABA/glycine neurons that hyperpolarize spinal motoneurons, producing REM-like paralysis during wakefulness. This mechanism explains how cataplexy represents a pathological intrusion of REM-atonia into conscious states and underscores the pivotal role of orexin neurons in stabilizing the dynamic interface between emotion, REM sleep regulation, and motor control.

The conceptual roots of narcolepsy lie in early clinicopathological observations from the encephalitis lethargica era, which implicated diencephalic regulatory circuits in the control of sleep and motor tone. Contemporary work reframes those historical insights through the lens of orexin (hypocretin) deficiency, integrating neurochemical, circuit, and immunological data into a coherent systems-level model (47, 79). Over the past two decades, the orexin system has emerged as the central hub, and more recent studies have refined our understanding of how loss of orexin signaling destabilizes arousal networks, disrupts REM gating, and perturbs emotion–motor integration (**Figure 4**).

### 11.1 Neurochemical foundations

#### 11.1.1 Orexin (hypocretin)

Orexin neurons in the lateral hypothalamus function as high-order coordinators of arousal, sleep-wake transitions, reward processing, autonomic regulation, and energy balance. They innervate and excite histaminergic, monoaminergic (noradrenergic, dopaminergic, serotonergic) and cholinergic nuclei, thereby stabilizing wakefulness and suppressing inappropriate intrusion of REM-related phenomena (47, 80). Orexin neurons fire most robustly during active wake with high motor tone and goal-directed behavior, providing a “persistence” signal to cortical and subcortical arousal systems (81).

The link between orexin loss and narcolepsy is strongly supported. Postmortem human studies show loss of up to 90–95% of orexin-producing neurons in most NT1 brains, accompanied by markedly reduced CSF orexin-A levels in more than 90% of patients (46, 48). Animal models replicate this relationship: mice lacking orexin peptides or harboring targeted inactivation of orexin receptor-2 signaling develop EDS, cataplexy-like events, and REM instability (12). Human data indicate that the severity of EDS and frequency of cataplexy correlate with the degree of CSF orexin depletion, whereas partial deficiencies are more often associated with NT2 phenotypes and may forecast later emergence of cataplexy (41, 42, 47).

Downstream monoaminergic relays are critical effectors of orexin action. Orexinergic input to locus coeruleus noradrenergic neurons helps sustain cortical activation and attention; disruption of this axis contributes to sleep-wake fragmentation and impaired vigilance (81). Similarly, orexin modulation of dorsal raphe serotonergic neurons influences both REM suppression and cataplexy: manipulating serotonergic tone in orexin-deficient mice suppresses cataplexy at the cost of increasing REM sleep, underscoring a delicate balance between anti-cataplectic and REM-promoting forces (54). Recent receptor-mapping work in mouse and human tissues shows dense expression of orexin receptors in brainstem arousal centers, limbic nodes, and the basal forebrain, supporting a broad neuromodulatory reach that extends beyond simple wake promotion (82).

Epigenetic studies now suggest that not all orexin neurons in NT1 are necessarily anatomically destroyed. Human postmortem data indicate promoter hypermethylation of the *HCRT* gene, reduced chromatin accessibility, and preserved expression of neighboring peptides within orexin-lineage neurons—consistent with epigenetic silencing of functionally “dormant” orexin cells in at least a subset of patients (44, 54). This distinction between neuronal loss and silencing has major implications for reversibility and regenerative strategies.

#### 11.1.2 Histaminergic and other systems

Histaminergic neurons in TMN are among the principal downstream targets of orexin. Their firing and histamine release peak during wake, and experimental suppression reduces arousal (80). In NT1, CSF histamine and tele-methylhistamine levels show heterogeneous changes, likely reflecting a blend of compensatory upregulation, altered metabolism, and immune influences (47). Postmortem analyses of the human TMN have described a higher number of HDC–immunoreactive neurons in NT1 compared with controls; however, the magnitude of this difference varies across studies and depends on methodological factors, including tissue processing, immunolabeling protocols, and whether unbiased stereological counting or marker-based cell identification was used (47, 74). These findings are consistent with compensatory histaminergic plasticity, but they should be interpreted cautiously, as increased HDC immunoreactivity may reflect increased enzyme expression or phenotypic shifts rather than a true increase in neuron number.

Clinically, the efficacy of the H_3_ receptor inverse agonist pitolisant in improving wakefulness and reducing cataplexy validates the functional significance of histaminergic modulation in human narcolepsy (75). Orexin neurons also co-release glutamate, dynorphin, and proteins such as the neuronal activity–regulated pentraxin, and they intersect with other sleep-regulatory systems, including melanin-concentrating hormone neurons and the prostaglandin D pathways, although the precise contribution of these cotransmitters in human narcolepsy remains incompletely defined (80).

### 11.2 Circuit and neurophysiological mechanisms

#### 11.2.1 Network destabilization and sleep-wake instability

Orexin neurons send dense projections to canonical arousal nodes, including the locus coeruleus, dorsal raphe, ventral tegmental area, TMN and basal forebrain, providing a tonic excitatory “gain control” that stabilizes wakefulness and supports sustained behavioral engagement (79). In animal models, intracerebroventricular orexin or selective OX_2_ receptor agonists prolong wake episodes and suppress cataplexy, while genetic or pharmacologic disruption of orexin signaling produces abrupt transitions into sleep and REM (83, 84).

Deficiency of orexin signaling during sleep has specific consequences for REM architecture. A 2023 PNAS study showed that mice lacking orexin receptor signaling selectively during sleep exhibit fragmented, unstable REM sleep and abnormal REM transitions, providing direct evidence that orexin’s role extends into the sleeping brain and is essential for maintaining consolidated REM structure (83, 84). In humans, NT1 patients display marked sleep-wake fragmentation, shortened REM latency, multiple SOREMPs and increased state transitions, which correlate with the degree of orexin depletion (47, 75).

#### 11.2.2 REM atonia dysregulation, cataplexy, and REM intrusion

A balance between REM-promoting and REM-inhibiting circuits orchestrates REM sleep. In the intact brain, glutamatergic neurons in the sublaterodorsal nucleus (SLD) drive GABAergic and glycinergic interneurons in the medulla and spinal cord that suppress motoneuron activity, producing the physiological atonia of REM sleep. REM-on SLD activity is restrained in wake and non-REM sleep by inhibitory inputs from ventrolateral periaqueductal gray and lateral pontine tegmentum, supplemented by serotonergic dorsal raphe and noradrenergic locus coeruleus tone (54, 79).

In narcolepsy, cataplexy is best conceptualized as pathological activation of REM-atonia circuitry during wake, often triggered by positive emotions. However, cataplexy is not simply REM atonia intruding into wakefulness: its pharmacology, EEG signatures, and clinical phenomenology overlap only partially with those of REM sleep (54, 79). Sleep paralysis, hypnagogic hallucinations and RBD similarly reflect disordered gating of individual REM components across sleep-wake transitions.

#### 11.2.3 Emotion–motor coupling and the amygdala–hypothalamus axis

Why do strong positive emotions so reliably trigger cataplexy? Functional MRI and EEG–fMRI studies in NT1 demonstrate altered activation and connectivity among the hypothalamus, amygdala, medial prefrontal cortex (mPFC) and brainstem during emotional stimuli, with abnormal coupling within limbic–motor networks (85, 86). In orexin-deficient mice, central amygdala (CeA) GABAergic neurons have emerged as key drivers of emotion-induced cataplexy: selective activation of CeA GABAergic neurons markedly increases cataplexy, while optogenetic inhibition reduces reward-promoted attacks (54, 79).

These CeA neurons project densely to the ventrolateral periaqueductal gray and the lateral pontine tegmentum, where they suppress REM-inhibiting circuits, and to the dorsal raphe and other monoaminergic structures, thereby facilitating activation of atonia pathways during wake (54, 79). Lesions or functional disruption of the amygdala reduce cataplexy in animal models, underscoring its causal role (54, 87). In healthy conditions, orexin provides excitatory drive to REM-inhibiting structures and modulates amygdala-prefrontal interactions, buffering the impact of emotional stimuli. In NT1, loss of orexin removes this stabilizing influence, allowing emotional inputs to disinhibit REM-atonia circuits and precipitate cataplexy.

Upstream cortical control is also implicated. The mPFC regulates emotional responses and exerts top-down control over the amygdala. In orexin-deficient mice, mPFC neurons show hypersynchronous theta activity during cataplexy, and optogenetic suppression of mPFC activity reduces attack frequency, suggesting that aberrant prefrontal–limbic synchronization contributes to attack generation (54). In humans, scalp EEG and source-imaging studies reveal increased theta and altered default-mode and salience network dynamics during cataplexy-like episodes, consistent with dysfunction in cortico-limbic control of motor tone (86).

#### 11.2.4 REM instability and large-scale network reorganization

Beyond discrete attacks, orexin deficiency promotes broader REM instability. Loss of orexin modulation during sleep disinhibits REM generators at inappropriate times, producing short, fragmented REM episodes and increased REM transitions (84). NT1 neuroimaging studies demonstrate structural and functional alterations in the hypothalamus, thalamus, amygdala, hippocampus and frontal cortex, together with disrupted resting-state connectivity in networks involved in salience, emotion and executive control (50, 86). Advanced MRI and PET approaches also point to reorganization of dopaminergic and limbic circuits, potentially contributing to reward, psychiatric and cognitive manifestations (48).

A recent study showed that NT1 patients exhibit abnormal physiological brain pulsations and CSF dynamics compared with healthy controls, suggesting that orexin may influence glymphatic function and metabolic waste clearance—raising provocative questions about long-term neurodegenerative risk (88).

#### 11.2.5 Integrative view and translational implications

Taken together, the pathophysiology of narcolepsy can be conceptualized as a multi-level cascade:

a. Genetic and immune-mediated disruption or epigenetic silencing of orexin neurons, driven by HLA- and T-cell–restricted autoimmunity and modulatory epigenetic mechanisms (44, 46, 54);

b. Destabilization of arousal networks, with failure of orexin-dependent excitatory drive leading to fragmented wakefulness, impaired vigilance and REM intrusions (48, 79, 84);

c. Pathological gating of REM-atonia circuits under emotional drive, centered on aberrant CeA–periaqueductal–brainstem–mPFC interactions, producing cataplexy, sleep paralysis and related phenomena (54, 79, 87);

d. Network reorganization and compensation, including histaminergic and monoaminergic plasticity, large-scale connectivity changes and potential alterations in brain fluid dynamics (50, 74, 88).

Translational work increasingly targets these mechanisms. Orexin receptor agonists—particularly highly selective OX_2_R agonists—have shown efficacy in improving wakefulness and reducing cataplexy in both animal models and early human trials, offering a mechanistically grounded replacement strategy (49, 83, 88). In parallel, regenerative approaches, including orexin cell transplantation and gene transfer into defined hypothalamic or limbic targets, have demonstrated proof-of-concept for restoring motor–arousal synchrony and reducing cataplexy in rodents (12).

Key unanswered questions remain. We do not yet know the proportion of orexin neurons that are epigenetically silenced rather than destroyed, nor the extent to which they can be reactivated. The interplay between ongoing immune attack, epigenetic repression and structural plasticity is poorly understood. Finally, it is unclear whether circuit-level interventions—such as targeted neuromodulation of limbic or brainstem nodes—can durably restore network stability in humans. As molecular, circuit and imaging tools converge, narcolepsy is gradually shifting from a purely symptomatic sleep disorder toward a candidate for truly disease-modifying, mechanism-based therapies.

## 12 Treatment

Management of narcolepsy is inherently multidimensional. Optimal care combines patient education, behavioral strategies, psychosocial support, and long-term medical follow-up rather than relying on a single pharmacological agent (75, 89). Treatment decisions must account for age, occupational demands, pregnancy and lactation, and comorbidities such as depression, obesity, cardiovascular disease, restless legs syndrome, periodic limb movements, RBD and sleep-disordered breathing (75); available interventions and key considerations are outlined in **Table 3**. Clinicians typically monitor sleepiness using the Epworth Sleepiness Scale, the Maintenance of Wakefulness Test (MWT), and increasingly patient-reported outcome measures that quantify quality of wakefulness and health-related quality of life (90, 91).

**Table 3:** Current treatment of narcolepsy.

Domain	Interventions (with dosage)*	Approval Status*	Evidence & Considerations
Non-pharmacological approaches	Scheduled naps; regular nocturnal sleep; sleep hygiene; psychoeducation for patient/family/school/workplace; psychotherapy/CBT; self-help groups; exercise programs; strategic caffeine use; weight control and balanced diet with reduced carbohydrate load	—	Foundational first-line for all patients. Improve coping, functioning, and adherence. Psychoeducation reduces stigma and facilitates accommodations (e.g., protected naps, avoiding night shifts). Exercise and weight control address elevated cardiometabolic risk. Limited controlled trials but broad expert consensus.
EDS	Modafinil 100–400 mg/day; Armodafinil 150–250 mg/day; Methylphenidate 10–60 mg/day; Mixed amphetamine salts or dexamphetamine 10–60 mg/day; Solriamfetol 75–150 mg/day (up to 300 mg)	Modafinil: FDA/EMA (NT1/NT2). Armodafinil: FDA only. Methylphenidate: FDA (NT1/NT2), EMA IR (NT1). Amphetamines: FDA (mixed salts), dexamphetamine approved in Germany/Switzerland. Solriamfetol: FDA 2019; EMA approved for narcolepsy-related EDS in 2024 (per trial data)	Modafinil/armodafinil remains first-line with favorable tolerability and low abuse potential. Classical stimulants provide strong wake promotion but have higher cardiovascular and abuse risks. Solriamfetol shows robust ESS/MWT gains and sustained benefits over 52 weeks; the most common AEs include insomnia, headache, and nausea. Extension and meta-analytic data demonstrate durable functional and QoL improvements.
Cataplexy	Sodium oxybate 4.5–9 g/night divided; ON-SXB 6–9 g once nightly; LXB equivalent dosing; Antidepressants (venlafaxine 37.5–300 mg; fluoxetine 20–60 mg; clomipramine 10–50 mg; citalopram 10–75 mg); Pitolisant 4.5–36 mg/day	Sodium oxybate: FDA (NT1/NT2), EMA (NT1). LXB: FDA/EMA. Antidepressants: clomipramine approved in Germany; otherwise off-label. Pitolisant: EMA (NT1/NT2), FDA pediatric review ongoing	Sodium oxybate is highly effective for both EDS and cataplexy, with improvements emerging over months. ON-SXB eliminates nocturnal redosing and improves adherence; early efficacy seen at lower doses (4.5–6 g). LXB reduces the sodium load by ~92% and supports weight reduction—both important for cardiometabolic risk. Antidepressants suppress cataplexy rapidly but may worsen RBD/restless legs. Pitolisant provides class I evidence for cataplexy reduction, with favorable tolerability and minimal abuse potential.
Other symptoms (sleep fragmentation, hallucinations, sleep paralysis)	Sodium or low-sodium oxybate; antidepressants (as above)	Oxybate: FDA/EMA	Oxybate improves nocturnal sleep continuity and reduces hallucinations and sleep paralysis, but may worsen sleep-disordered breathing in OSA-susceptible individuals. Antidepressants may aggravate RBD or periodic limb movements.
Oxybate formulations	ON-SXB (FT218/LUMRYZ) 6–9 g once nightly; LXB (XYWAV) 4.5–9 g nightly divided	ON-SXB: FDA 2023. LXB: FDA/EMA	ON-SXB replaces twice-nightly dosing, increasing convenience and adherence. LXB reduces sodium exposure with preserved efficacy and may promote weight loss. Phase 4 and real-world data support improved QoL and cardiometabolic outcomes.
Pitolisant	4.5–36 mg/day	EMA (NT1/NT2); FDA pediatric and adult review in progress	Long-term data show sustained benefit on EDS and cataplexy with minimal abuse potential. A pediatric RCT (2023–2024) supports approval for≥6 years. Neutral effect on weight; non-controlled substance.
Orexin receptor agonists	Oveporexton (TAK-861, oral): 8-week trial dosing; Danavorexton (TAK-925, IV): short-infusion trial dosing; TAK-994 (oral): program discontinued	None approved; Phase 2/3 trials ongoing	These agents directly target the underlying orexin deficit. Oveporexton (NEJM 2025) produced large improvements in MWT, ESS, and cataplexy with no hepatotoxicity. Danavorexton rapidly normalizes wakefulness and reduces sleep-wake fragmentation, with additional peri-operative applications (restoring respiration and consciousness). TAK-994 showed strong efficacy but was halted due to hepatotoxicity. Represents a therapeutic class specifically designed to target the underlying orexin signaling deficit.
Pediatric therapy	Sodium oxybate (as above); ON-SXB 6–9 g nightly; Pitolisant 4.5–36 mg; stimulants, modafinil, antidepressants (mostly off-label)	Sodium oxybate FDA ≥7 years; LXB FDA/EMA ≥7 years; pitolisant EMA ≥6 years	Pediatric management is increasingly evidence-based. Pitolisant offers a non-stimulant alternative with favorable tolerability. Weight gain, early puberty, and behavioral issues require multidisciplinary care.
Combination regimens	Oxybate + modafinil; oxybate + pitolisant; oxybate + stimulants	No formal approvals	Commonly used in practice to optimize control of EDS, nocturnal sleep, and cataplexy. Evidence remains limited; treatment must be individualized.
Pregnancy & lactation	Minimizing or discontinuing medication is preferred; modafinil, oxybate, and selected antidepressants are sometimes continued with caution	No formal approvals	Registry data raise concerns for teratogenicity (modafinil) and neonatal effects; decisions must be individualized, ideally with pre-conception counselling and close obstetric oversight.
Immunotherapy (experimental)	IVIG; high-dose corticosteroids; plasmapheresis; monoclonal antibodies (natalizumab, alemtuzumab)	Not approved	Only case reports and small series suggest partial benefit when used early after disease onset. No RCT since 2015 shows durable efficacy. Consider only for hyperacute onset or overlapping inflammatory disease.

*Approval status refers to narcolepsy indications at the time of writing; dosing ranges may require individual titration. CBT, cognitive behavioral therapy; EDS, excessive daytime sleepiness; ESS, Epworth Sleepiness Scale; MWT, Maintenance of Wakefulness Test; RBD, REM sleep behavior disorder; OSA, obstructive sleep apnea; NT1/NT2, narcolepsy type 1/type 2; IR, immediate release; QoL, quality of life; RCT, randomized controlled trial; FDA, U.S. Food and Drug Administration; EMA, European Medicines Agency; ON-SXB, once-nightly sodium oxybate; LXB, low-sodium oxybate; IVIG, intravenous immunoglobulin.

### 12.1 Non-pharmacological approaches

Non-pharmacological strategies form the foundation of care. Clinicians should work with patients to implement regular nocturnal sleep schedules, optimize sleep hygiene and schedule one or two brief, strategic daytime naps to offset irresistible sleep attacks (75). Psychoeducation—directed at patients, families, employers and schools—reduces stigma and facilitates reasonable accommodations (e.g. protected nap times, avoidance of night shifts, safe-driving plans) (92). Exercise programs, judicious caffeine use and weight-control strategies are particularly relevant given the elevated cardiometabolic risk profile in narcolepsy (90, 92). Cognitive-behavioural therapy and participation in patient support groups may improve coping, mood and treatment adherence (75).

### 12.2 Symptomatic pharmacological approaches

Pharmacotherapy complements but does not replace lifestyle measures. Drug therapy complements, but does not substitute for, behavioral measures. Historically, clinicians relied on classical stimulants and antidepressants; contemporary practice now draws on modafinil/armodafinil, solriamfetol, oxybate formulations, pitolisant and, increasingly, orexin receptor agonists (75, 93).

### 12.3 Excessive daytime sleepiness

#### 12.3.1 Modafinil and armodafinil

Modafinil remains a first-line wake-promoting agent in many guidelines, typically prescribed at 100–400 mg/day in divided morning and early-afternoon doses; armodafinil is used at 150–250 mg once daily (89). Randomized trials and meta-analyses show that both agents improve Epworth scores and MWT latencies with a generally acceptable tolerability profile, though they can increase headache, nausea and anxiety (94). Their relatively low abuse potential compared with amphetamines supports long-term use, provided cardiovascular and psychiatric status are monitored (95).

#### 12.3.2 Traditional stimulants

Methylphenidate (often 10–60 mg/day in divided doses) and mixed amphetamine salts are typically reserved as second- or third-line wake-promoting therapies when modafinil or solriamfetol are insufficient or contraindicated (75). Network meta-analyses suggest robust wake-promoting effects but at the cost of higher rates of sympathomimetic adverse events and abuse potential (93).

#### 12.3.3 Solriamfetol

Solriamfetol, a dopamine–noradrenaline reuptake inhibitor, represents a more recent addition. The pivotal phase 3 trial (NCT02348593) randomized 236 adults with narcolepsy to solriamfetol 75, 150 or 300 mg once daily versus placebo for 12 weeks and demonstrated dose-dependent improvements in MWT latency and Epworth scores, with significant benefits at 150 and 300 mg (96). A 52-week extension showed sustained functional and quality-of-life gains with a safety profile dominated by dose-related insomnia, headache and nausea (91). A systematic review of four clinical trials confirmed robust improvements in EDS with acceptable tolerability (97). These data underpin regulatory approvals in North America and Europe for EDS in narcolepsy, typically at 75–150 mg/day, titrated up to 300 mg as tolerated (91).

### 12.4 Cataplexy

#### 12.4.1 Sodium and low-sodium oxybate

Oxybate formulations remain the cornerstone of cataplexy treatment. Traditional sodium oxybate, given in two nightly doses totaling 6–9 g/night, reduces both EDS and cataplexy frequency, with maximal benefits often emerging after 3–6 months (98). The phase 3 REST-ON trial evaluated once-nightly sodium oxybate (FT218; ON-SXB) at 6, 7.5 and 9 g given as a single bedtime dose. In adults with NT1 or NT2, ON-SXB significantly improved MWT, Epworth scores, and weekly cataplexy rates compared with placebo, with effect sizes comparable to or greater than those of twice-nightly formulations (99, 100). *Post-hoc* analyses support meaningful improvements in both NT1 and NT2 populations and show early efficacy as soon as week 1 at 4.5–6 g (99).

Low-sodium oxybate (LXB; calcium–magnesium–potassium oxybates) reduces sodium load by ≈92% while maintaining clinical efficacy. In phase 3 and extension studies in narcolepsy with cataplexy, LXB preserved reductions in cataplexy frequency and EDS while contributing to modest weight loss—a relevant advantage in a population at risk for obesity (92). LXB is now approved in the United States for EDS or cataplexy in patients ≥7 years with narcolepsy and for idiopathic hypersomnia in adults (92, 101). Real-world data suggest that many patients prefer switching from high-sodium to low-sodium formulations to mitigate cardiovascular risk, provided titration and expectations are carefully managed (98). However, the high cost of sodium oxybate and its restricted regulatory availability in many regions remain significant barriers to equitable global access (102).

#### 12.4.2 Antidepressants

Tricyclic antidepressants (e.g., clomipramine, imipramine) and serotonin–noradrenaline reuptake inhibitors (e.g., venlafaxine, duloxetine) suppress cataplexy by enhancing monoaminergic tone, particularly during REM transitions (103). These drugs remain widely used, especially when oxybate is unavailable or contraindicated, although no contemporary randomized trials have secured formal regulatory indications (75). Clinicians must weigh anticholinergic burden, QT interval prolongation and propensity to exacerbate RBD or restless legs syndrome (75, 104).

#### 12.4.3 Pitolisant

Pitolisant, a histamine H_3_ receptor antagonist/inverse agonist, reduces both EDS and cataplexy by enhancing histaminergic activity and downstream arousal circuits. The pivotal HARMONY-CTP trial demonstrated that pitolisant (up to 40 mg/day) significantly reduced weekly cataplexy rates and improved EDS versus placebo in adults with severe NT1 (105). Network meta-analyses and long-term extension studies confirm that pitolisant demonstrates efficacy comparable to modafinil or oxybate for EDS, with a favorable abuse-liability profile similar to placebo (93, 106). In 2023, a phase 3 trial in 110 children aged 6–17 years showed that pitolisant improved narcolepsy symptoms, with a safety profile similar to that in adults, supporting EMA approval for use in pediatric NT1 and NT2 from age 6 years (107).

### 12.5 Other symptoms and nocturnal sleep

Sodium and low-sodium oxybate improve nocturnal sleep continuity, reduce awakenings and may attenuate hypnagogic hallucinations and sleep paralysis, though they can worsen sleep-disordered breathing in susceptible individuals and require cautious use in patients with untreated obstructive sleep apnea (82, 88). Antidepressants can aggravate RBD and restless legs syndrome, necessitating careful polysomnographic monitoring when parasomnias emerge or worsen (75). There is growing interest in whether solriamfetol and pitolisant can improve cognitive performance and daytime functioning beyond their effects on sleepiness, with initial data in populations with hypersomnolence and obstructive sleep apnea demonstrating improvements in attention, processing speed, and work productivity (91, 97, 108).

### 12.6 New therapeutic advances

**Figure 5:**
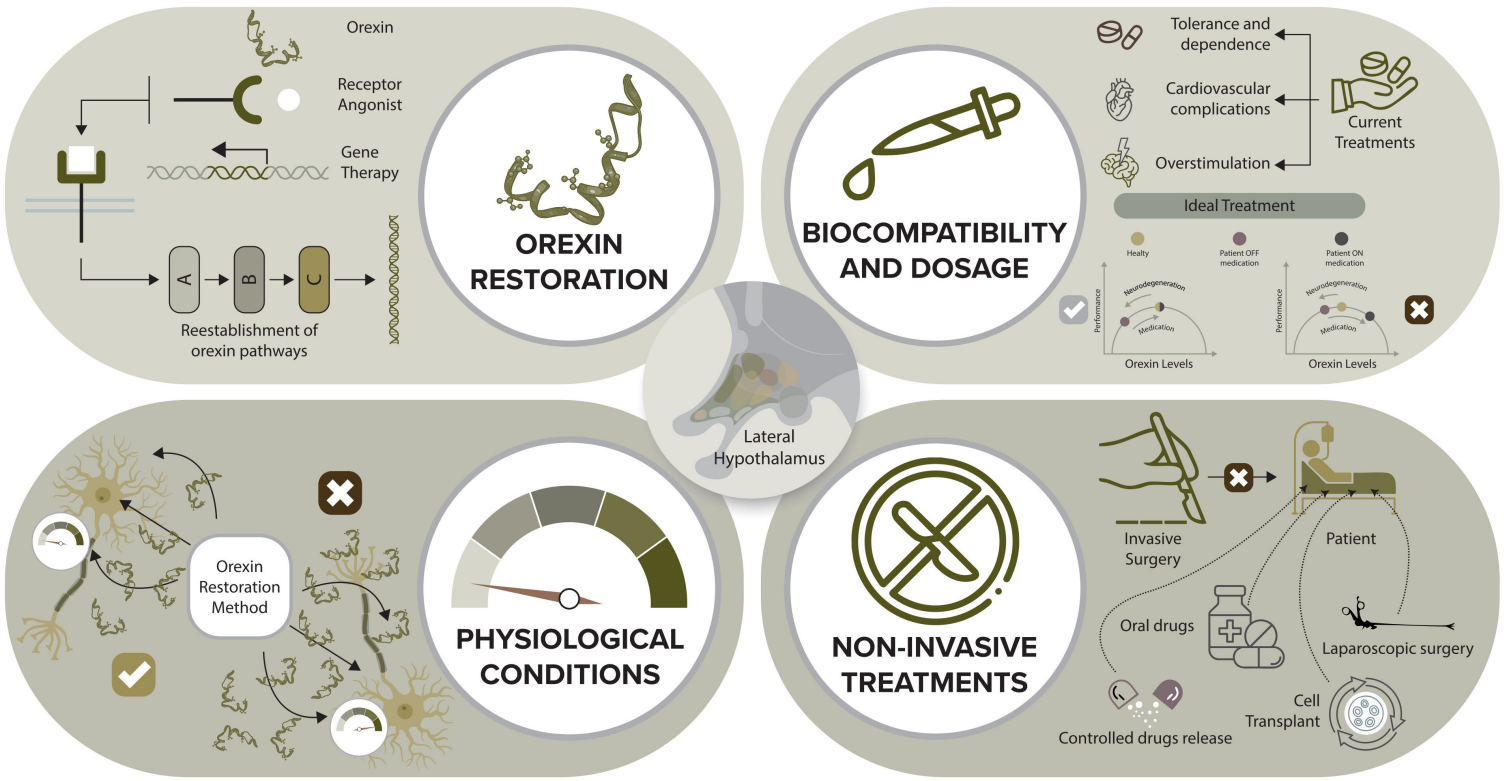
Therapeutic strategies to restore orexin signaling and advance mechanism-based care in narcolepsy. Orexin restoration (top left): Therapeutic strategies include exogenous orexin peptide administration, selective orexin receptor agonists, and gene therapy designed to reinstate orexin expression and downstream signaling across arousal networks. The goal is stepwise restoration of physiological orexin pathways, enabling sustained wakefulness and improved REM sleep regulation. Physiological conditions (bottom left): Mechanism-targeted interventions aim to approximate endogenous orexin dynamics rather than relying solely on symptomatic stimulation. Conceptual comparisons illustrate how therapeutics engineered to normalize orexin tone may stabilize arousal thresholds and reduce state intrusions. Biocompatibility and dosage (top right): Current medications can be limited by cardiovascular side effects, tolerance, dependence, or overstimulation. Next-generation therapies must optimize dosing, receptor selectivity, and long-term safety to ensure durable benefit without excessive sympathetic activation. Non-invasive treatments (bottom right): Future strategies emphasize minimally invasive delivery, including orally bioavailable orexin receptor agonists, controlled-release platforms, and potentially orexin cell replacement using neurosurgical approaches refined to reduce operative risk. Collectively, these interventions represent a transition from symptomatic management to mechanism-based therapy, with the potential to alter the disease trajectory by restoring the orexin system.

Beyond established symptomatic therapies, novel and future interventions are being developed to restore orexin signaling and modify the disease trajectory in narcolepsy (**Figure 5**).

#### 12.6.1 Oxybate formulations

The therapeutic landscape for oxybate has diversified. ON-SXB offers once-nightly dosing with sustained efficacy for both EDS and cataplexy, addressing the practical challenge of nocturnal redosing and potentially improving adherence (99). LXB provides comparable symptomatic control with dramatically reduced sodium exposure and favorable longer-term cardiometabolic implications (92, 98). Phase 4 data from the DUET study program and real-world cohorts continue to refine titration strategies and document weight loss and improved quality of life in both narcolepsy and idiopathic hypersomnia (109, 110).

#### 12.6.2 Pitolisant

Beyond adult NT1/NT2 indications, the 2023–2024 pediatric trial expanded pitolisant’s regulatory footprint to children ≥6 years, providing a non-sodium, non-controlled alternative to stimulants and oxybate (107). Long-term observational studies indicate sustained benefits for EDS and cataplexy, with a neutral or favorable profile regarding weight gain and abuse potential (111, 112).

#### 12.6.3 Solriamfetol

Longitudinal extension studies up to 52 weeks show that solriamfetol maintains wake-promoting efficacy with stable dosing (75–300 mg/day) and consistent improvements in work productivity and quality-of-life indices (91). Contemporary systematic reviews position solriamfetol as a first-line or early second-line agent for EDS in narcolepsy, particularly when modafinil is ineffective or poorly tolerated (97).

#### 12.6.4 Orexin receptor agonists

a. Mechanism-based therapy targeting the primary orexin deficit represents a major advance of the past five years. Oral and injectable orexin receptor 2 (OX_2_R) agonists now provide proof of concept and early clinical efficacy.

b. TAK-994. In a phase 2 NEJM trial, the first oral OX_2_R agonist, TAK-994, produced substantial improvements in MWT, Epworth scores and weekly cataplexy frequency over 8 weeks in adults with NT1, with many participants approaching normative wakefulness ranges (10). However, clinically significant hepatotoxicity led to early termination of the program, underscoring the need for safer agents (113).

c. Danavorexton (TAK-925). Danavorexton is an injectable OX_2_R-selective agonist with rapid wake-promoting effects. Studies in orexin/ataxin-3 narcoleptic mice and in human NT1/NT2 patients show dose-dependent increases in MWT latency to ceiling values, reductions in sleep-wake fragmentation, and suppression of cataplexy-like episodes (49, 114). Phase 1 and translational studies also demonstrate that danavorexton can reverse opioid-induced respiratory depression and anesthetic sedation without compromising analgesia, highlighting potential perioperative applications (115).

d. Oveporexton (TAK-861). A key advance is the development of oveporexton, a next-generation oral OX_2_R agonist. In the 2025 phase 2 NEJM trial (NCT05687903), oveporexton, administered once or twice daily for 8 weeks in adults with NT1, produced large, dose-dependent improvements in MWT latency, Epworth scores, and weekly cataplexy rates, with many participants achieving near-normal wakefulness and substantial cataplexy suppression (11). Importantly, no hepatotoxicity or major visual adverse events were observed, and safety profiles were favorable across doses (11). Ongoing phase 3 trials report similarly robust efficacy, supporting OX_2_R agonism as a therapy designed to address the core orexin deficit in narcolepsy directly.

Collectively, these agents validate the concept that direct restoration of orexin signaling can normalize core narcoleptic symptoms more effectively than downstream symptomatic agents, and they open the door to mechanism-based disease modification.

### 12.7 Pediatric treatment

Pediatric treatment has historically extrapolated from adult data. Sodium oxybate received FDA approval for EDS and cataplexy in patients 7–17 years, and LXB now extends that indication while reducing sodium exposure (92). The recent pitolisant phase 3 trial in children 6–17 years, with or without cataplexy, demonstrated clinically meaningful improvements in narcolepsy symptom scores and acceptable tolerability, leading to EMA approval and offering an oral, non-controlled alternative (107). Stimulants and modafinil remain widely used off-label, but only oxybate formulations and pitolisant currently carry pediatric indications (75). Pediatric management must also address rapid weight gain, early puberty and neurobehavioral issues, often requiring close collaboration with endocrinology and psychiatry.

### 12.8 Special considerations

Combination regimens—such as oxybate plus modafinil, or oxybate plus pitolisant—are common in practice to balance nocturnal consolidation, daytime wakefulness and cataplexy control, although controlled data on specific combinations remain sparse (75, 98). LXB may reduce weight yet occasionally exacerbate mood symptoms; antidepressants may improve mood but worsen RBD or restless legs (92, 104).

During pregnancy and lactation, clinicians generally aim to minimize or discontinue narcolepsy medications where feasible. Registry and observational data suggest that modafinil, oxybate, and certain antidepressants may pose teratogenic or neonatal risks; individualized risk–benefit assessment, pre-conception counselling, and close obstetric collaboration are essential (75). For patients in high-risk occupations (e.g., professional drivers, pilots, healthcare workers), fitness-for-duty evaluations and structured return-to-work plans are critical.

### 12.9 Immunotherapy

Given the strong autoimmune signature of NT1, immunomodulatory strategies have attracted interest, particularly in very early disease. Case reports and small series have described partial benefit from intravenous immunoglobulin, high-dose corticosteroids, plasmapheresis, or B- and T-cell–directed monoclonal antibodies, including natalizumab and alemtuzumab, when administered within weeks to months of onset (46). However, no randomized controlled trial since 2015 has demonstrated durable efficacy, and immunotherapy remains an experimental, off-label approach reserved for select patients with hyperacute presentation, concurrent systemic autoimmunity or overlapping inflammatory CNS disease (46). The absence of validated biomarkers of early immune activation and the difficulty of identifying a narrow therapeutic window continue to limit progress.

### 12.10 Outlook

Treatment of narcolepsy has entered a qualitatively new era, marked by an increasingly stratified evidence base that now supports both symptomatic control and mechanism-targeted intervention (**Table 4**). Once, low-sodium oxybate formulations have refined symptomatic management and reduced sodium burden; pitolisant and solriamfetol offer mechanistically distinct wake-promoting options with favorable long-term profiles; and, critically, orexin receptor agonists now deliver the first targeted therapy that addresses the core molecular deficit rather than downstream consequences (10, 11, 24, 33, 75, 101, 107, 112). The next decade will determine whether combination strategies—integrating orexin agonists, oxybate, wake-promoting agents and, potentially, early immunomodulation—can not only control symptoms but also modify disease trajectory, preserve residual orexin neurons and reshape the long-term prognosis of narcolepsy.

**Table 4:** Expert consensus summary of evidence and recommendations for current narcolepsy treatments.

Intervention	Primary Target	Certainty of Evidence*	Strength of Recommendation*	Rationale based on provided evidence
Non-pharmacological: sleep hygiene, scheduled naps, psychoeducation, CBT, exercise, weight control	EDS, cataplexy support, QoL	Low	Strong	Universal clinical consensus; limited controlled data; consistent behavioral benefit; no RCT evidence.
Modafinil / Armodafinil	EDS	High	Strong	Multiple RCTs, meta-analyses; consistent improvement in ESS/MWT; good safety; long-term evidence.
Traditional stimulants (methylphenidate, amphetamines)	EDS	Moderate	Conditional	Strong efficacy, but safety and abuse liability reduce certainty; network meta-analysis evidence; fewer contemporary RCTs.
Solriamfetol	EDS	High	Strong	Phase 3 RCT + 52-week extension + systematic review of 4 trials; consistent functional and QoL gains; well-characterized safety.
Sodium oxybate (twice nightly)	Cataplexy, EDS, nocturnal sleep	High	Strong	Robust RCT data; long-term effectiveness; established in multiple populations.
Once-nightly sodium oxybate (ON-SXB)	Cataplexy, EDS	High	Strong	Phase 3 RCT with dose-dependent efficacy; comparable or superior to divided dosing; good early response.
LXB	Cataplexy, EDS	High	Strong	Phase 3 + extension evidence; comparable efficacy with major sodium reduction; real-world cardiometabolic benefit.
Antidepressants (TCAs, SNRIs, SSRIs)	Cataplexy	Low to Moderate	Conditional	Effective suppression of cataplexy but no modern RCTs; risk of worsening RBD/restless legs; mostly observational evidence.
Pitolisant	EDS, cataplexy	High	Strong	Pivotal RCT (HARMONY), meta-analyses, long-term data; pediatric RCT supports broader use; good safety and no abuse liability.
Orexin receptor agonists (oveporexton, danavorexton; TAK-994)	Core disease mechanism	Moderate to High (varies)	Conditional (pending approval)	TAK-861 (oveporexton) shows robust RCT efficacy without hepatotoxicity → High certainty. Danavorexton has strong translational and early clinical evidence but limited long-term data → Moderate certainty. TAK-994 halted due to toxicity → Very low certainty.
Pediatric treatments: oxybate, pitolisant	EDS, cataplexy	High	Strong	RCTs leading to regulatory approvals for ages ≥6 (pitolisant) and ≥7 (oxybate); consistent safety and efficacy.
Combination therapy (oxybate + modafinil/pitolisant/stimulants)	EDS + cataplexy	Moderate	Conditional	Strong clinical usage but few controlled trials; evidence mainly from observational cohorts and expert consensus.
Pregnancy & lactation management	Safety	Very low	Conditional	Sparse registry/observational data; no RCTs; risk–benefit individualized.
Immunotherapy (IVIG, steroids, plasmapheresis, monoclonals)	Early autoimmune phase	Very low	Conditional/Weak	Only case reports and small series; no durable efficacy in trials since 2015; experimental.

*Certainty of evidence and strength of recommendation were derived from expert consensus using a GRADE-informed framework, based on study design hierarchy, consistency of findings, effect magnitude, and regulatory-level evidence. This table does not represent a formal GRADE assessment. CBT, cognitive behavioral therapy; EDS, excessive daytime sleepiness; QoL, quality of life; RCT, randomized controlled trial; ESS, Epworth Sleepiness Scale; MWT, Maintenance of Wakefulness Test; ON-SXB, once-nightly sodium oxybate; LXB, low-sodium oxybate; TCAs, tricyclic antidepressants; SNRIs, serotonin–noradrenaline reuptake inhibitors; SSRIs, selective serotonin reuptake inhibitors; RBD, REM sleep behavior disorder; IVIG, intravenous immunoglobulin.

## 13 Discussion

The discovery of the orexin system transformed the conceptual landscape of narcolepsy, yet contemporary understanding continues to outpace clinical practice. Current evidence positions narcolepsy, particularly narcolepsy type 1, as an immune-mediated hypothalamic encephalopathy in which selective or functional loss of orexin neurons destabilizes arousal networks and alters emotional–motor integration (46, 51, 75). Despite this shift in mechanistic clarity, diagnostic criteria remain anchored in a narrow symptom framework defined by cataplexy and sleep-onset REM episodes. The resulting misalignment between biological insight and clinical implementation has perpetuated diagnostic delay, hindered early recognition, limited therapeutic innovation and allowed substantial underdiagnosis across populations.

Important gaps in the evidence base impede progress. Epidemiological estimates remain uncertain, with prevalence varying dramatically across global populations, likely reflecting differences in genetic susceptibility, infectious exposures and environmental triggers (36). Methodological inconsistencies further complicate interpretation. Diagnostic reliance on the multiple sleep latency test and overnight polysomnography fails to capture the heterogeneity of narcolepsy and performs poorly in children, secondary cases and atypical phenotypes (75). Although cerebrospinal fluid orexin-A measurement remains the strongest biomarker, immunoassays lack standardization and exhibit substantial inter-laboratory variability, limiting their application outside specialized centers (73). Meanwhile, the identification of autoreactive CD4^+^ and CD8^+^ T cells reactive to orexin-related peptides provides strong evidence of an autoimmune pathogenesis (3–5), yet definitive causal pathways have not been mapped longitudinally in humans.

Therapeutic progress has been similarly constrained. Contemporary treatment remains dominated by symptomatic strategies that provide partial relief but do not modify the underlying disease process. Modafinil, amphetamines, pitolisant, solriamfetol and oxybate formulations improve excessive daytime sleepiness and cataplexy but often fail to address the broader hypothalamic dysfunction that contributes to psychiatric, metabolic and autonomic comorbidities (75). Treatment adherence is variable, and therapeutic decisions rarely incorporate immune signatures, neuroimaging findings or genetic risk profiles. Immunomodulatory treatments have shown encouraging results in isolated reports but have not been tested rigorously in controlled trials (46). Comorbidities such as depression, obesity, metabolic dysregulation, autonomic instability and chronic pain remain under-recognized in routine care, despite robust evidence of their contribution to disability and reduced quality of life.

The field now stands at an inflection point. A modern clinical framework must transcend rigid NT1–NT2 dichotomies and instead adopt an integrated phenotype–biomarker–mechanism model. Early clinical suspicion should prioritize pathological sleepiness, vigilance disruption, and atypical emotional–motor phenomena, particularly in children, in whom rapid weight gain, behavioral dysregulation, and facies cataplectica often precede classic symptoms (116). Biomarker integration should extend beyond CSF orexin quantification to include high-sensitivity LC–MS peptide assays, immunological profiling, HLA genotype, hypothalamic and limbic neuroimaging signatures and emerging peripheral markers of T-cell activation (42, 50). Diagnostic classification should reflect mechanistic diversity, distinguishing autoimmune orexin neuron loss from partial orexin dysfunction, secondary hypothalamic injury and hereditary narcolepsy syndromes. This stratification would allow symptomatic therapy in established orexin-deficient disease, immunomodulatory therapy in early or evolving phenotypes and regenerative or receptor-targeted therapy in chronic states.

Integrated management must reflect the full systemic footprint of narcolepsy. Psychiatric, metabolic and autonomic dysfunction are intrinsic components of hypothalamic pathology and require systematic evaluation rather than peripheral attention. This approach reframes narcolepsy as a multisystem disorder of hypothalamic networks rather than a primary sleep disorder.

Scientific advances now enable realistic consideration of disease-modifying strategies. Small-molecule orexin receptor 2 (OX_2_R) agonists represent a pivotal development. Recent phase 2 data for oveporexton (TAK-861) demonstrate robust improvements in excessive daytime sleepiness, cataplexy and patient-reported quality of life, without the hepatotoxicity that halted the development of earlier agents (11). Intravenous danavorexton (TAK-925) provides further validation of receptor agonism as a therapeutic class, producing rapid wake-promoting effects in early-phase trials (49). In parallel, immunological studies identifying autoreactive T cells, T-cell receptor specificity, and systemic immune signatures raise the possibility that early immune-modulating interventions may prevent, attenuate or delay orexin neuron loss (45, 61). Longitudinal, pre-symptomatic cohorts will be essential to determining when autoimmunity begins, how rapidly orexin neurons are lost or silenced and which patients might benefit from targeted early intervention.

The field also moves closer to pre-symptomatic diagnosis. The convergence of HLA-associated risk, autoimmune signatures, prodromal symptoms such as early weight gain and REM instability, and machine-learning models trained on large datasets suggests that identification of at-risk individuals may soon be feasible (36, 42). A shift from late recognition to early detection, and ultimately to prevention, is within conceptual reach.

The future agenda spans several domains. Epidemiology requires an updated, harmonized methodology to delineate true prevalence and clarify the distribution of narcolepsy spectrum disorders. Disease framing must recognize narcolepsy as a global hypothalamic disorder encompassing motor, cognitive, psychiatric, emotional, metabolic and autonomic systems. Etiological studies should integrate genetic, environmental and epigenetic layers, with attention to immune markers, infection-related triggers and comorbidity patterns. Mechanistic research must extend beyond orexin to dissect compensatory or maladaptive changes in histaminergic, monoaminergic and limbic–brainstem networks. Diagnostic development must advance beyond the MSLT toward high-resolution orexin assays, neurophysiological REM-instability markers, quantitative video-cataplexy analysis, neuromelanin and hypothalamic MRI, and multi-omics biomarker discovery supported by machine-learning approaches. Therapeutics require patient-centered endpoints, validated disease severity scales, and rigorous trials in children, pregnant individuals, and those with atypical phenotypes. Non-pharmacological interventions, including structured napping and dietary optimization, merit systematic evaluation. Finally, disease-modifying pathways—including orexin replacement, gene therapy, stem-cell approaches and early immune-targeted strategies—should be prioritized, particularly for individuals with evolving phenotypes or partial orexin dysfunction.

In sum, narcolepsy should be reconceptualized as a family of hypothalamic encephalopathies unified by orexin dysfunction and immune targeting but heterogeneous in phenotype, etiology and prognosis. A modern framework grounded in biomarkers, mechanistic stratification and multisystem care can close the gap between scientific knowledge and clinical practice. Such an approach positions narcolepsy as a model for neuroimmune disease and opens the possibility that, with continued mechanistic insight and early detection, progression—and perhaps even onset—may one day be preventable.
